# Salinity-driven stratification enhances riverine mercury export to the coastal ocean

**DOI:** 10.1016/j.ecss.2025.109595

**Published:** 2025-11-03

**Authors:** Roland P. Ovbiebo, Cathryn D. Sephus, Amina T. Schartup

**Affiliations:** Scripps Institution of Oceanography, University of California San Diego, USA

**Keywords:** Methylmercury, River discharge, Residence time, Estuary types, Biogeochemical transformations

## Abstract

Rivers transport 300 to 5000 Mg of mercury (Hg) annually to coastal oceans through estuaries, contributing 20–45% of total Hg input, with 100 to 1500 Mg reaching the open ocean. However, the impact of estuarine circulation and stratification on Hg transport and methylation remains uncertain despite their known influence on other metal exports. This study developed three models to assess Hg transformation under different salinity-driven stratification regimes—well-mixed, slightly stratified, and highly stratified—using data from the Chesapeake Bay (CPB) and Hudson River Estuary (HRE), U.S.A. Results show that stratification increases riverine Hg export by 19% in CPB and 20% in HRE, with shorter Hg residence times promoting faster export. Unstratified estuaries favor Hg burial in sediments due to longer residence times and increased particle settling. Seasonal river discharge variations further influence stratification, with higher discharge enhancing stratification and Hg export. Methylmercury (MeHg) production and export also respond to stratification, with slightly stratified conditions in CPB increasing MeHg production by 11.5% and export by 16.4%. As climate change is expected to intensify stratification in many estuaries, these findings suggest potential increases in Hg and MeHg export to coastal oceans.

## Graphical Abstract

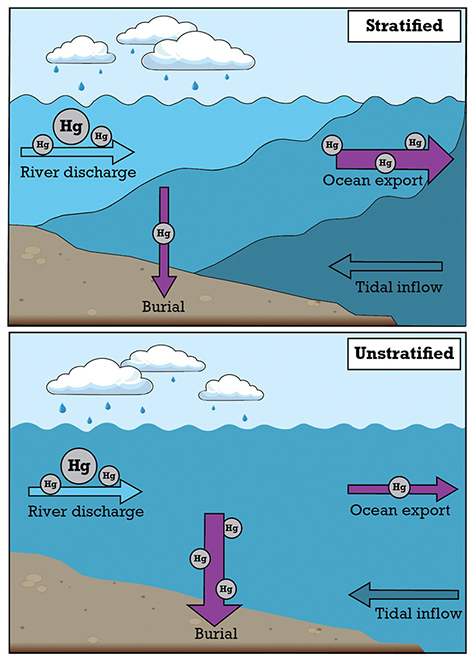

## 1. Introduction

Estuarine mercury (Hg) biogeochemical cycling is unique due to the dynamic mixing of riverine freshwater and saline ocean water; thus, understanding the estuarine processes that regulate Hg export to the ocean is important. These mixing processes influence Hg speciation, behavior, and transport across the land-ocean continuum, affecting regional and global Hg cycles (Fitzgerald et al., 2007; Mason et al., 1993). While much attention has historically been given to Hg deposited to the ocean through atmospheric processes, recent studies indicate that riverine sources contribute 1000 Mg of Hg to the coastal ocean annually (Liu et al., 2021), which is three times more than the 310 Mg of Hg deposited through atmospheric processes to the same area. Accurately quantifying the riverine flux of Hg is challenging, as it is affected by numerous factors, including variations in river discharge, sampling limitations, hydrodynamic processes, and the influence of suspended sediment and organic matter (Liu et al., 2021; Amos et al., 2014). Consequently, current global riverine Hg export estimates to the oceans vary widely, ranging from 300 to 5000 Mg annually (Liu et al., 2021; Amos et al., 2014; Cossa et al., 1996; Outridge et al., 2018).

The behavior and fate of contaminants within estuaries, including Hg, are influenced by the estuarine circulation and stratification patterns, which vary among estuary types (Costa et al., 2010; Iglesias et al., 2019; Papaslioti et al., 2024). Estuaries are typically classified into three main types based on stratification: well-mixed, slightly stratified, and highly stratified. These classifications reflect variations in vertical and horizontal water movement, significantly affecting how contaminants are retained, flushed, or deposited, which has implications for water quality and ecosystem health (Chen et al., 2023; Marsooli et al., 2018; Williamson and Morrisey, 2000). Within these stratification regimes, physical and hydrodynamic processes further impact Hg’s speciation into its inorganic divalent (Hg^II^) and elemental Hg (Hg^0^), and organic forms – mono- and di-methylmercury (MMHg and DMHg, respectively). In highly stratified estuaries, dense bottom water restricts vertical mixing, trapping Hg species in deeper waters and sediments, where microbial activity in anoxic sediments converts inorganic Hg into methylmercury (MeHg; sum of MMHg and DMHg), a bioaccumulating neurotoxicant (Carrasco et al., 2011; Chen et al., 2014; Harding et al., 2018), through various methylation processes (Mason et al., 1993; Chen et al., 2019; Merritt and Amirbahman, 2008; Schartup et al., 2013). In slightly stratified systems, vertical and horizontal mixing disperses Hg throughout the water column, and methylation processes can occur in the pycnocline (Mason et al., 1999), which is a layer within the water column where there is a rapid change in water density with depth caused by variations in salinity, temperature, or both. These systems also enhance Hg binding with organic matter, which promotes settling and subsequent methylation in the benthic sediment (Schartup et al., 2013; Chakraborty et al., 2015; Mazrui et al., 2016). In well-mixed estuaries, Hg species are more evenly distributed due to the absence of stratification, reducing localized accumulation between different salinity layers. However, strong tidal forces in these systems can resuspend Hg-containing sediments, releasing Hg into the water column, where it may undergo methylation if conditions permit (Heyes et al., 2004; Liu et al., 2023a).

Considering these dynamics, we propose that incorporating the distinct stratification characteristics of these estuarine types in modeling riverine Hg flux to the coastal ocean could help reduce uncertainties in current global Hg flux estimates. To address this, we develop three empirically constrained models of Hg cycling dynamics tailored to each stratification-specific estuarine system, aiming to refine estimates of riverine Hg discharge into the coastal ocean. We use these models to examine the role of salinity-driven stratification in modulating Hg speciation within a specific coastal framework. Our model was evaluated using observational data from the Chesapeake Bay (CPB) and Hudson River Estuary (HRE) in the United States. These two estuarine systems exhibit seasonal variability in stratification; CPB transitions from slightly stratified to well-mixed, and HRE transitions from highly stratified to slightly stratified, reflecting fluctuations in freshwater inflow and tidal mixing. By modeling Hg dynamics across these stratification scenarios, we offer new insights into the role of estuarine stratification in Hg transport and transformation, with implications for better estimates of riverine Hg contributions to the coastal ocean and an improved understanding of its global distribution.

## 2. Methodology

### 2.1. Study area description

The CPB, the largest estuary in the United States, stretches about 322 km from the Susquehanna River in Maryland to Cape Charles and Cape Henry in Virginia, with a surface area of about 11,600 km^2^ and a watershed that spans over 165,000 km^2^ (Supplementary Fig. S1). The Susquehanna River significantly affects salinity levels in the estuary, providing approximately 62% of the freshwater supply and directly feeding into the Bay’s main stem. Other significant rivers flowing into the Bay include the Potomac, James, York, and Rappahannock Rivers. With an average water depth of 7 m and an exchange rate of 8000 m^3^s^−1^ with the ocean (Austin, 2002), the estuary features a two-layer circulation: fresh, lighter water flows seaward on the surface, while denser, saltier water moves landward below. The pycnocline separates these layers, resulting in slightly stratified conditions with seasonal variation. The stratification is strongest in spring and mixing increases in fall due to seasonal changes in river discharge and wind-driven mixing, creating well-mixed conditions in the summer months. The estuary experiences tidal mixing, but large portions are brackish water (0.5–25 psu). Tidal forces are modest, rarely exceeding a 1 m range (Browne and Fisher, 1988), with wind and tidal mixing influencing the estuary salinity and residual circulation (Li et al., 2005; Wilson, 1988).

The HRE is a tidal estuary that spans 246 km from Battery at New York Harbor to the Federal Dam at Troy, located on the northeastern coast of the United States (Supplementary Fig. S1). It is much smaller in surface area (5700 km^2^) than the CPB, with a watershed of about 34,700 km^2^. The Hudson River is the primary water body, with smaller tributaries feeding into the estuary, such as the Mohawk Creek, Rondout Creek, and Esopus Creek. The estuary has an average depth of roughly 10 m with a mean tidal flow of around 12,040 m^3^s^−1^ (Cooper et al., 1988). It features a dynamic ecosystem shaped by freshwater inflows, tides, and varying salinity (5–30 psu), with tidal ranges reaching 2 m and peak velocities of 1 ms^−1^ (Warner et al., 2005). Freshwater from upstream flows into the Atlantic, while tidal forces push saltwater upstream, creating a salt-wedge or highly stratified estuary (National Oceanic and Atmospheric Administration, 2024). The estuary’s strong tides dominate the river’s flow patterns over much of its length. The HRE is more stratified than the CPB, with salinity extending up to 140 km from the Battery depending on freshwater flow (Wells and Young, 1992).

### 2.2. Model framework

**Fig. 1.**
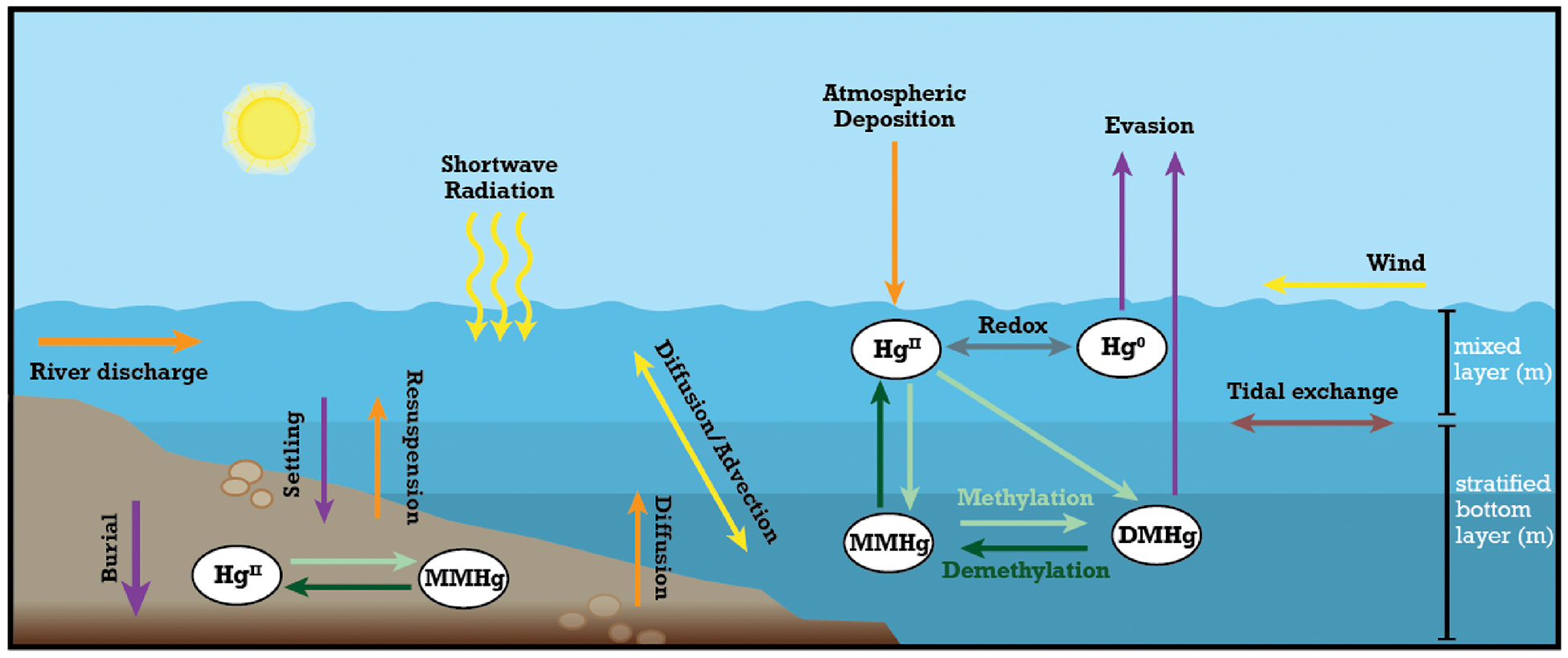
Conceptual diagram of estuarine mercury (Hg) cycling processes considered in the model. Each arrow color represents a distinct pathway in the Hg cycling process: orange arrows indicate external inputs into the water column, purple arrows show outputs from the water column, light green arrows represent methylation, dark green arrows depict demethylation, and gray arrow illustrate redox reactions among the major Hg species (divalent: Hg^II^, elemental: Hg^0^, monomethylmercury: MMHg, and dimethylmercury: DMHg). Brown arrows represent the bidirectional net tidal exchange (tidal inflow and ocean export), while yellow arrows highlight physical forcing and estuarine mixing mechanisms.

We constructed an empirically constrained mass budget for the four main Hg species based on the stratification type of estuaries building on Sunderland et al. (2010) in Python (version 3.12.2). Our model considers how the different physical, chemical, and hydrodynamic processes affect the speciation and transformation of Hg species in estuaries and what this means for the export of riverine Hg to the ocean and MeHg production in the water column and sediments (Fig. 1). The various processes and fluxes of Hg species captured in the model include (1) external inputs from river discharge, atmospheric deposition, and inflow of tidal water from the ocean, (2) chemical transformation through inorganic Hg^II^ and Hg^0^ redox reactions, methylation of Hg^II^ and MMHg, and demethylation of MMHg and DMHg, (3) advective and diffusive mixing in the water column and outflow into the ocean, (4) diffusion/bioturbation from sediment porewater to the overlaying water through the sediment-water interface, (5) settling of particulate Hg^II^ and MMHg in the water column and to sediments, the resuspension from and burial in benthic sediments, and (6) evasion of Hg^0^ and DMHg from the water surface through air-sea gas exchange (Fig. 1).

Fig. 1 illustrates Hg cycling within an estuary, highlighting the interactions between the atmosphere, water column, and sediment. Hg enters the estuary through river discharge, tidal inflow, and atmospheric deposition, undergoes transformations (methylation, demethylation, redox reactions), and is transported through diffusion, advection, settling, and resuspension processes. Hg can also evade into the atmosphere or be exported to the ocean. Physical factors like wind, shortwave solar radiation, and water currents also influence this cycling.

#### 2.2.1. Mass budget model

The concentration of Hg species in each system is based on a review of measured concentrations and fluxes from the literature (Table 1). The original datasets report concentration ranges and/or means, with distributions that are likely log-normal—a pattern commonly observed in environmental trace metal studies (Živković et al., 2025; Ott, 1990; Pacyna, 2020; Schuster et al., 2011). Therefore, we report geometric means and geometric standard deviations to more accurately reflect the central tendency and variability of the data. These values were used to initialize Hg species reservoir concentrations in the mass budget model (Supplementary Table S1–S2). Previous literature does not contain the measurements of all four species of Hg needed to model the biogeochemical, physical, and hydrodynamic processes affecting Hg cycling in the two estuaries. Therefore, we separated the total Hg (THg) and MeHg measurements into the four Hg species (Hg^II^, Hg^0^, MMHg, and DMHg). Many studies report MeHg concentrations without distinguishing between MMHg and DMHg. Because of the lack of data on DMHg in estuaries, we input that MMHg is 60% and DMHg is 40% of MeHg based on average ratios reported in marine studies (Bieser et al., 2023; Lehnherr et al., 2011; Mason and Sullivan, 1999; Munson et al., 2015). Sensitivity analyses (see Section 2.2.2) showed that varying the MMHg:DMHg ratio from 95:5 to 50:50 had a negligible impact (≤3%) on model outcomes. This limited influence is attributed to DMHg’s high volatility and degradation potential within the water column (Adams et al., 2024; West et al., 2022; Chen et al., 2024; West et al., 2020; He et al., 2025).

The Hg^II^ concentration was calculated as the difference between the THg and the sum of Hg^0^ and MeHg. The Hg^0^ concentration in CPB is assumed to be about the same concentration of dissolved gaseous Hg (the sum of Hg^0^ and DMHg), and is estimated to contain >90% of Hg^0^ in surface water (Mason et al., 1998). No published data for Hg^0^ concentrations in HRE exist, so we assume the concentration of Hg^0^ in the water column to be approximately 5% of THg concentrations based on literature, where Hg^0^ ranges between 1 and 9.3% in various estuarine environments (Mason et al., 1999; Baeyens and Leermakers, 1998; Benoit et al., 1998; Schartup et al., 2015). Mass budgets for each Hg species are used to create coupled first-order differential equations simulating chemical mass changes over time (Sunderland et al., 2010; Schartup et al., 2015; Soerensen et al., 2016). The model applies constant flow rates for tidal exchange (calculated as tidally averaged fluxes), river discharge, and atmospheric deposition (Supplementary Table S3). Riverine THg loading is estimated as the product of mean river discharge and observed Hg concentrations from the literature. To simplify and isolate the effects of stratification, riverine Hg concentration remains constant across different discharge conditions. Consequently, changes in riverine Hg flux in the model reflect variations in discharge rate rather than concentration. These external THg inputs—tidal input, river discharge, and atmospheric deposition—and outputs—ocean export, atmospheric evasion, and sediment burial—drive the model to reach a steady state. The model uses a 12-hour time step for biogeochemical transformations (methylation, demethylation, redox), with rate constants derived from literature. See Supplementary Tables S4–S16 for a detailed description of how the fluxes of the different hydrodynamic and Hg species biogeochemical processes are calculated.

#### 2.2.2. Model validation and sensitivity analysis

Model performance was conducted by comparing the predicted THg and MeHg ocean export with the observed concentrations of THg and MeHg in the CPB and HRE. The results indicate that the model predicts estuarine Hg cycling within ±20% of the observed values across systems, supporting its general applicability. A sensitivity analysis comparing model outcomes using both geometric and arithmetic means to initialize Hg reservoir sizes showed no differences, indicating that the model is robust to initial concentration assumptions and primarily governed by flux dynamics and internal transformation processes.

**Fig. 2.**
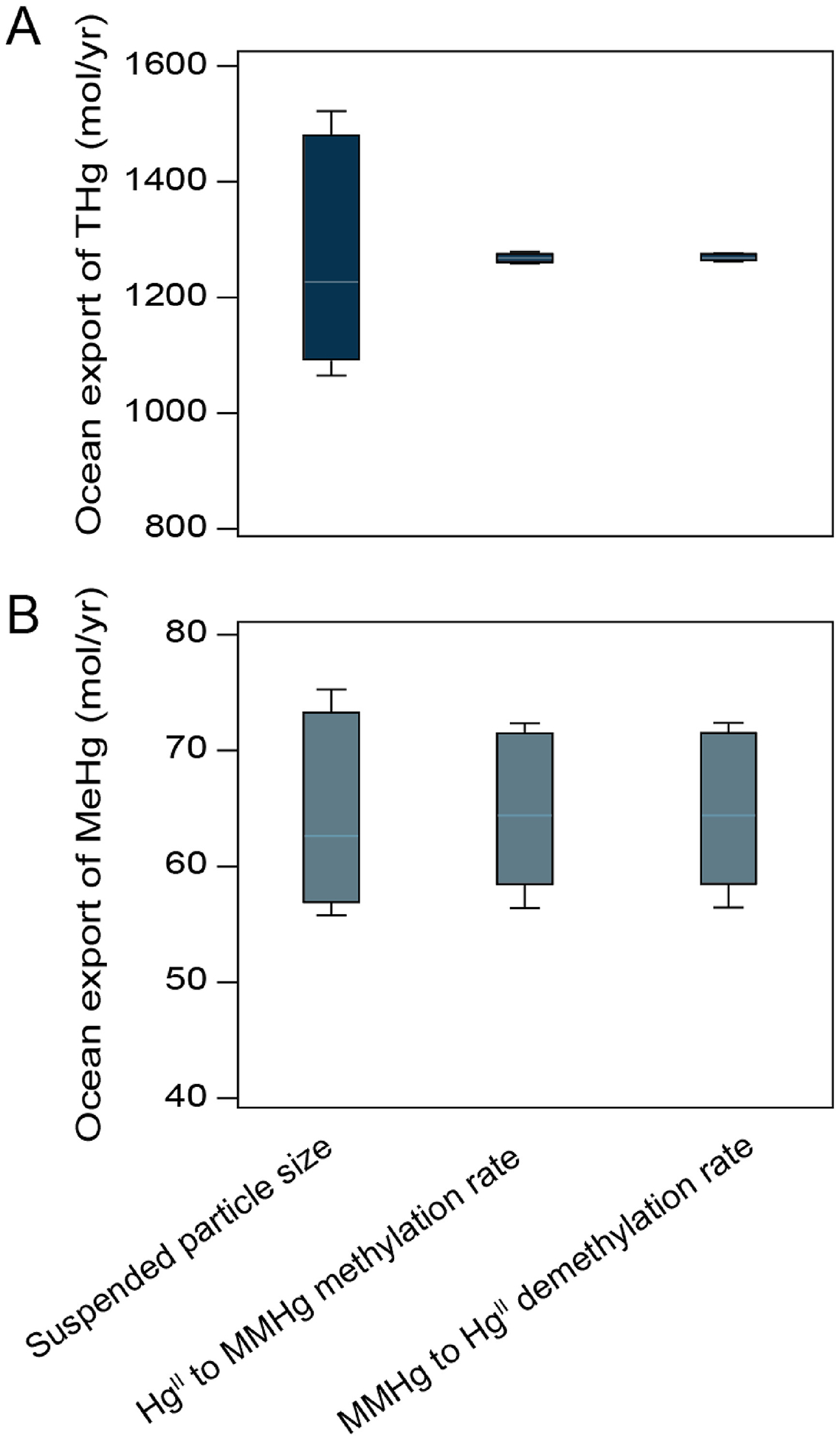
Variation in the amount of (A) THg and (B) MeHg exported to the ocean due to ±15 % change in key Hg biogeochemical transformation parameters in Chesapeake Bay. Each box-and-whisker plot represents the range of variability in THg and MMHg export resulting from individual parameter adjustments during the one-at-a-time (OAT) sensitivity analysis.

A one-at-a-time (OAT) sensitivity analysis was performed to assess the robustness of the model. This involved adjusting several key parameters—such as the methylation rates of Hg^II^ (0.0023–0.0031 day^−1^, (Lehnherr et al., 2011; Despins et al., 2023; Monperrus et al., 2007; Schartup et al., 2025), demethylation rates of MMHg (0.031–0.042 day^−1^, (Monperrus et al., 2007; Bouchet et al., 2013; Soerensen et al., 2018), suspended particle size (7.2–9.8 μm, (Chesapeake Bay Program, 2024; Gibbs et al., 1994; Liu et al., 2023b; Restrepo et al., 2024; Schubel, 1969), and MMHg and DMHg partition ratios (40:60, 50:50, 70:30, and 30:70 (Bieser et al., 2023; Lehnherr et al., 2011; Mason and Sullivan, 1999; Munson et al., 2015; Adams et al., 2024), within ranges supported by existing literature. A ±15% change in both the rates of methylation and demethylation, as well as in suspended particle size, resulted in less than a 20% variation in the modeled export of THg and MeHg to the ocean (Fig. 2). In contrast, a ±15 % variation in parameters such as MeHg speciation ratios (MMHg and DMHg), sediment-water diffusion coefficients, erosion rates, and burial rates had minimal impact (≤5%) on model outputs. This indicates that while the model outputs show moderate sensitivity to biogeochemical transformation rates, they are less sensitive to assumptions regarding MeHg speciation partitioning and other key parameters. Error bars in Fig. 2 represent deviations from baseline parameter values used in model simulations.

### 2.3. Stratification and residence time

The stratification of the estuaries was used to separate each estuary’s water column into different compartments and estimate the sizes of Hg reservoirs and residence times in each system (Supplementary Table S4–S6). To identify the stratification class of each estuary, we use the estuary’s basin-wide average vertical salinity data from Xu et al. (2012) and the New York City Department of Environmental Protection (New York City Department of Environmental Protection, 2015) to calculate the stratification parameter (*Δ*). We chose the salinity scheme method to estimate the stratification because there were multiple spatial salinity measurements along the estuary transect (Shen et al., 2022). *Δ* is computed as the tidally average salinity difference ratio between the surface (*S*_*sal*_) and bottom water (*B*_*sal*_) to the depth-averaged salinity (*A*_*sal*_) (Eq. (1)) (Shen et al., 2022; Hansen and Rattray, 1965).


1 $$\Delta =\frac{B_{sal}-S_{sal}}{A_{sal}}$$

When *Δ* is less than 0.1, it is classified as well-mixed; when *Δ* is between 0.1 and 1, it is slightly/partially stratified, and if larger than 1, it is highly/strongly stratified (Shen et al., 2022; Hansen and Rattray, 1965).

**Fig. 3.**
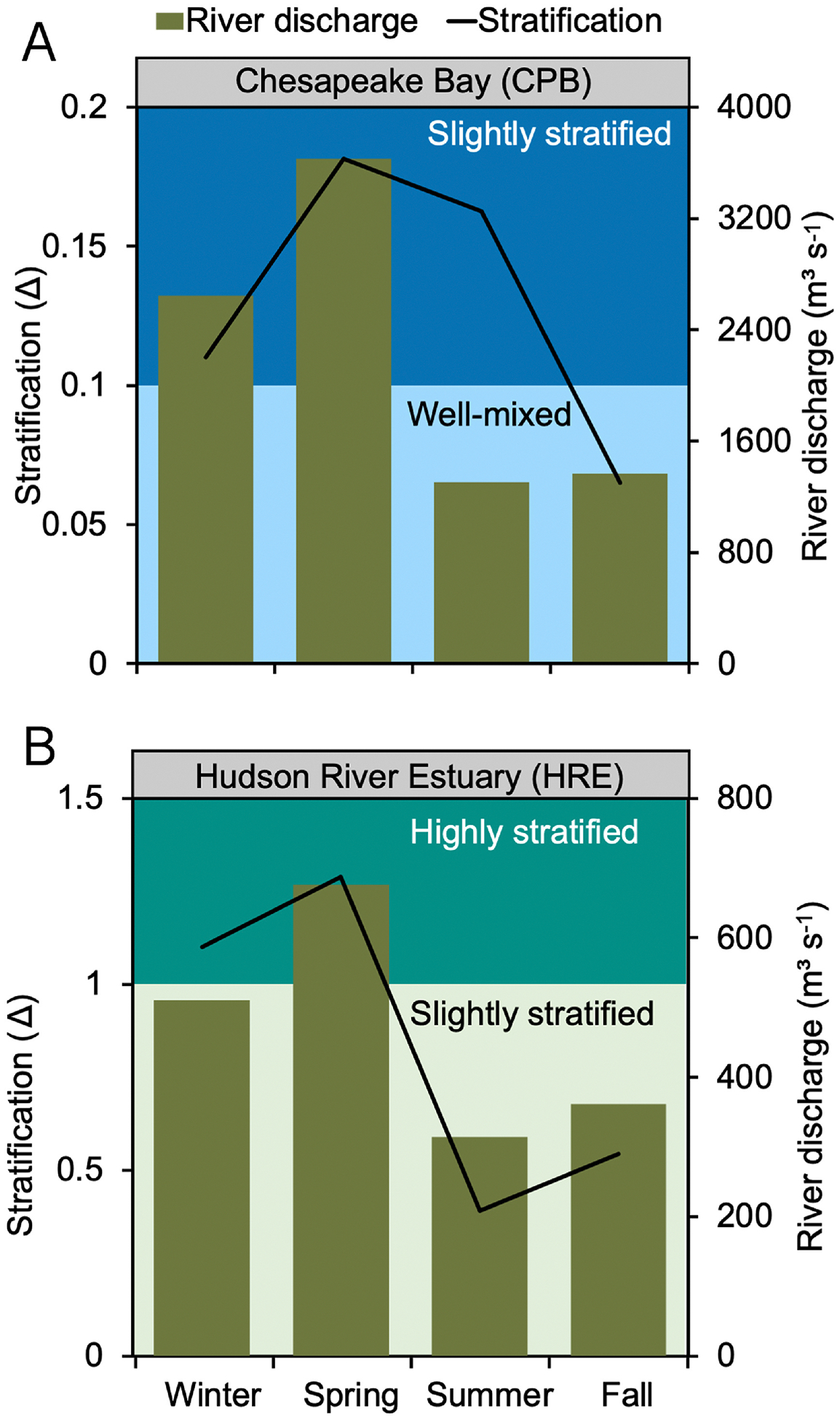
Basin-wide long-term seasonal variation in water column stratification and river discharge in (A) Chesapeake Bay (CPB) and (B) Hudson River Estuary (HRE). In both systems, the black lines represent the calculated stratification parameter, and the green bars show the average river discharge across four seasons: winter (December–February), spring (March–May), summer (June–August), and fall (September–November). The background shading indicates different levels of stratification, with light blue representing well-mixed conditions and dark blue representing slightly stratified conditions in CPB, while in HRE, light green shows slightly stratified conditions and dark green indicating highly stratified conditions.

The CPB stratification class varies seasonally from slightly stratified during the period coinciding with higher river inflow (>2600 m^3^s^−1^) to well-mixed estuary under low river flow conditions (<1400 m^3^s^−1^) (Fig. 3A). The system is more stratified in the winter (*Δ* = 0.11, December–February) and spring (*Δ* = 0.18, March–May). The stratification persists into the summer (*Δ* = 0.16, June–August) as the surface water warms up and transitions into well-mixed conditions in the fall (Δ = 0.07, September–November) as river outflow decreases.

The stratification types in HRE range from highly to slightly stratified. The strongest stratification occurs at intermediate salinities (13.7–14.9 psu) during the winter (*Δ* = 1.1) and spring (*Δ* = 1.29) due to higher river outflow (>500 m^3^s^−1^) and increased precipitation runoff into the estuary from its 34,700 km^2^ watershed area (Fig. 3B). The highly stratified conditions (*Δ* > 1) in the HRE are not solely due to increased freshwater inflow. The freshwater input rate must exceed the tidal mixing rate for stratification to persist. This balance can result from strong freshwater inflows, reduced tidal mixing, or a moderate combination of both influences, leading to the observed stratification (Geyer and Ralston, 2011a).

After determining the stratification type of each estuary, we used a hydrological budget equation (Eq. (2)) to estimate the seasonal depth of each compartment, which we classified as the mixed surface layer and stratified bottom layer. The calculations for the surface mixed layer and stratified bottom layer depths in both highly and slightly stratified systems incorporated the estuary’s total freshwater volume, surface area, and mean central depth (Supplementary Table S5). This approach enabled us to estimate the water volume in each system, which is necessary for calculating Hg residence time. Monthly river discharge data for the CPB and HRE were sourced from the USGS Water Data at the river mouths (Susquehanna, Potomac, Rappahannock, York, and James Rivers) and Green Island (near Troy Dam) (U.S. Geological Survey, 2024), respectively (Fig. S1).

2 $$S_{dep}=W_{dep}-M_{dep}$$

where *M*_*dep*_ is the mixed surface layer depth (m), *W*_*dep*_ is central channel average depth (m), and *S*_*dep*_ is the stratified bottom layer depth (m).

The time spent by Hg species in each system was determined using the water residence time in the estuary. The residence time (*τ*) in days was calculated using the freshwater method (Lemagie and Lerczak, 2014; Sheldon and Alber, 2006; Dyer, 1973). This method uses the water volume (*Vol*_*w*_), freshwater fraction (*FWF*), and freshwater inflow rate (*FW*_*fl*_) to estimate the estuary turnover time (Eq. (3) and Supplementary Table S6). This calculation was done for the 12 months of the year based on changes in the monthly freshwater inflow and salinity.


$$\tau =Vol_{w}*FWF/FW_{fl}$$

While initially developed for system dominated by tidal exchange (i. e. low flowing rivers). This method was successfully applied in previous studies across a broad range of estuarine conditions, including moderate-to high-flow river systems. For example, Lemagie and Lerczak (2014) applied the freshwater fraction method in Yaquina Bay and found consistent results with the particle tracking model, total exchange flow, and tidal prism approaches. Similarly, Shen et al. (2022) used the method to evaluate residence time in 360 USA estuaries, and Huang (2007) applied it in a small estuary of North Bay, USA, with <50 m^3^s^−1^ freshwater input.

We calculate that seasonally the residence times in the CPB range from 63 to 280 days, whereas those in the HRE are shorter, ranging from 12 to 20 days. These values are consistent with findings from previous studies in the two estuaries. The longer residence times in CPB can be attributed to its larger size and lower flushing rates, while the shorter residence times in HRE are due to its smaller size and strong tidal flushing dynamics (Geyer and Chant, 2006).

## 3. Results and discussion

### 3.1. Water column stratification impacts riverine Hg flux to the coastal ocean and Hg removal in estuarine systems

We compare two estuary model simulations: one that is stratified, where the water column is divided into layers based on the salinity gradient, and another that is unstratified, where the salinity is uniform throughout the entire water column. Both models use the same physical, hydrodynamic, and Hg transformation processes to evaluate how stratification affects Hg removal in the estuaries and, ultimately, the amount of Hg that reaches the coastal ocean.

**Fig. 4.**
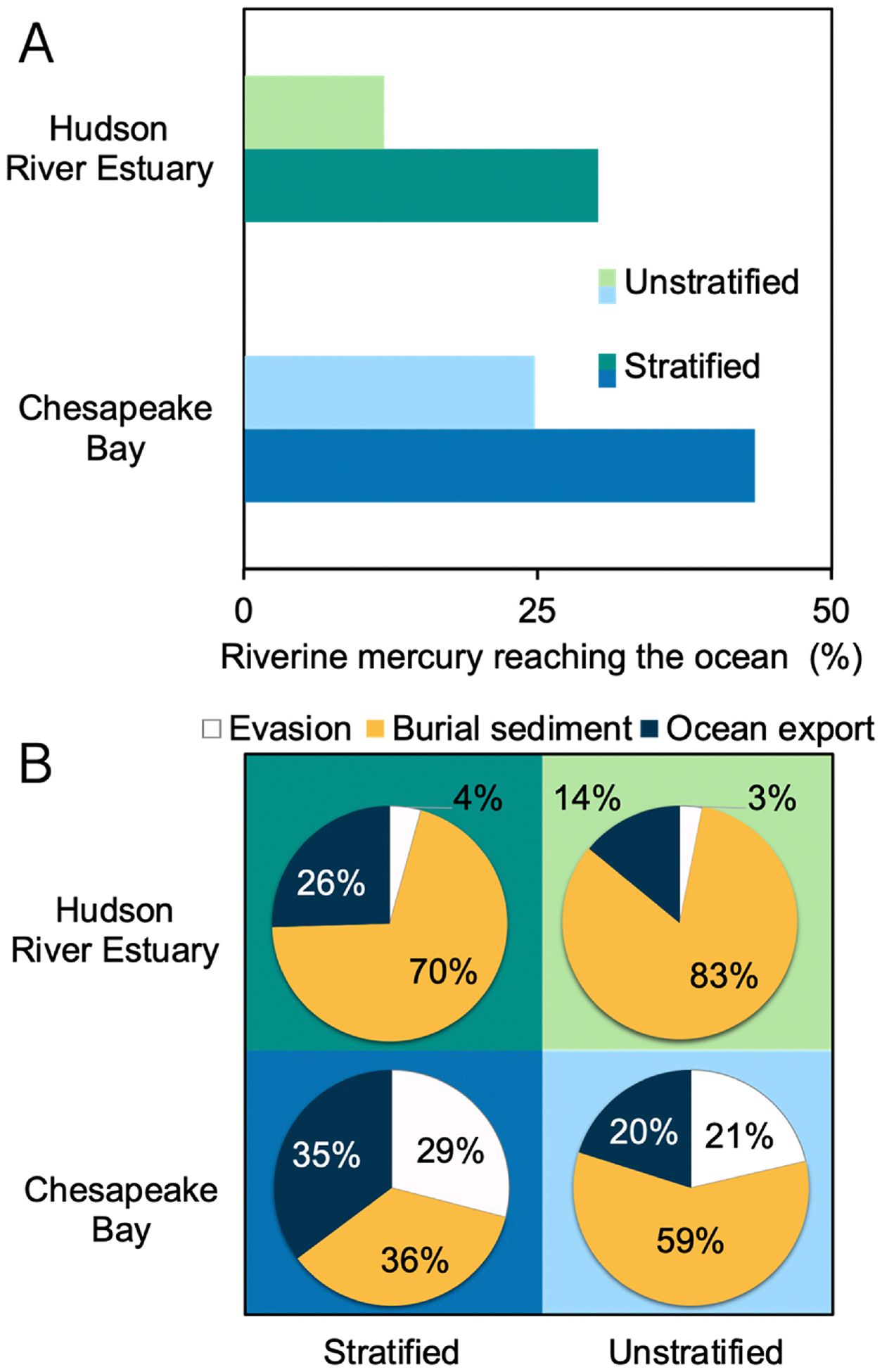
Effect of stratification on mercury (Hg) removal processes in the Chesapeake Bay and Hudson River Estuary. (A) Illustrates the total Hg (THg) percentage in river discharge reaching the coastal ocean. (B) The relative contribution of THg removal processes from stratified and unstratified estuaries. The light colors in the bar chart in Figure A and the background shading in the pie chart in Figure B represent unstratified estuaries, and the dark colors represent stratified estuaries. The color-coded segments in the pie charts represent different processes; the white color represents the evasion of gaseous Hg to the atmosphere, the yellow color represents the burial of THg in the sediment, and the dark blue color represents the export of THg from the estuary to the ocean.

Model results indicate that stratification enhances the export of Hg from rivers to the ocean. This trend is seen for both systems modeled in Fig. 4A, where we observe a 19% increase in Hg export in the CPB and 20% in the HRE as the system becomes more stratified. These results align with prior studies; for example, Mason et al. (1999) reported that 29% of the riverine Hg is exported to the ocean from CPB, which overlaps with our findings (25–44%). However, the prior study did not include stratification in estimating this Hg export. To our knowledge, no comparable analysis has been performed on the HRE. Our model suggests that the development of a pycnocline is key to controlling Hg export in stratified systems. In a stratified system, riverine THg remains in the surface water above the pycnocline, resulting in a shorter residence time within the estuary, and is more readily exported to the ocean.

We used the model to evaluate how water column stratification changes the Hg removal processes; these findings are summarized in Fig. 4B. We see that when a system is unstratified, the fraction of THg removed by burial in sediments increases. While stratification can vertically trap particle-bound Hg in the water column due to density gradients, unstratified systems typically exhibit lower flushing rates, resulting in longer overall residence times for Hg in the estuary. This extended residence time facilitates greater settling of particulate Hg and its subsequent sequestration in sediments. Our findings regarding the higher fraction of THg buried in unstratified systems align with other studies that assumed well-mixed conditions in their modeling of Hg in estuaries. These studies have reported that over 70% of riverine Hg is ultimately buried in estuarine sediment (Amos et al., 2014; Liu et al., 2023a; Buck et al., 2015; Meng et al., 2019; Molina et al., 2023; Zhang et al., 2015; Jia et al., 2021). We also see that evasion of Hg decreases by 23 and 13% in CPB and HRE, respectively, when the systems are unstratified (Fig. 4B). The observed changes can largely be attributed to longer residence time and the absence of a pycnocline. The longer residence time leads to a higher settling of Hg in unstratified conditions, leaving less Hg in the water column and decreasing the pool available for evasion. The absence of pycnocline in the unstratified system enhances tidal mixing of the large surface riverine Hg pool delivered to the estuary surface with the entire water column, leaving a lower concentration of Hg in the surface water for evasion. In unstratified estuaries, the mixing of riverine freshwater with seawater results in a shoaling of the euphotic depth, which occurs due to particles in the water that decrease the intensity of ultraviolet solar radiation. As a result, the euphotic depth decreases by 6% in CPB and by 32% in HRE, lowering the production and evasion of volatile Hg^0^. Our model results show that the absence of stratification in the estuary water column will increase the amount of Hg buried in estuarine sediment while decreasing evasion to the atmosphere and export to the coastal ocean.

While our model results indicate that estuarine stratification enhances Hg export to coastal oceans, a substantial fraction of THg entering the estuary is retained through burial and biogeochemical transformation under both stratified and unstratified states. These processes sequester Hg within estuarine sediments or convert it to gaseous forms lost to the atmosphere, reducing its downstream flux. The sedimentary reservoir, in particular, represents a long-term sink that can limit bioavailable Hg within the water column (Amos et al., 2014; Zhang et al., 2015). Given that estuaries serve as nursery habitats for many fish and invertebrates (Lewis et al., 2020; Hughes et al., 2014), the dynamics of Hg retention are equally important for understanding exposure risk and ecosystem health.

### 3.2. Hg removal processes in estuaries respond to seasonal variability in Hg sources and stratification type

In the previous section, we tested how estuaries respond to changes in stratification while keeping conditions constant over a year. However, the presence and strength of stratification vary by estuary and season. For example, CPB and HRE transition to more stratified states as river discharge increases (Fig. 3). Here, we model how seasonal variations in stratification influence Hg cycling in HRE and CPB.

**Fig. 5.**
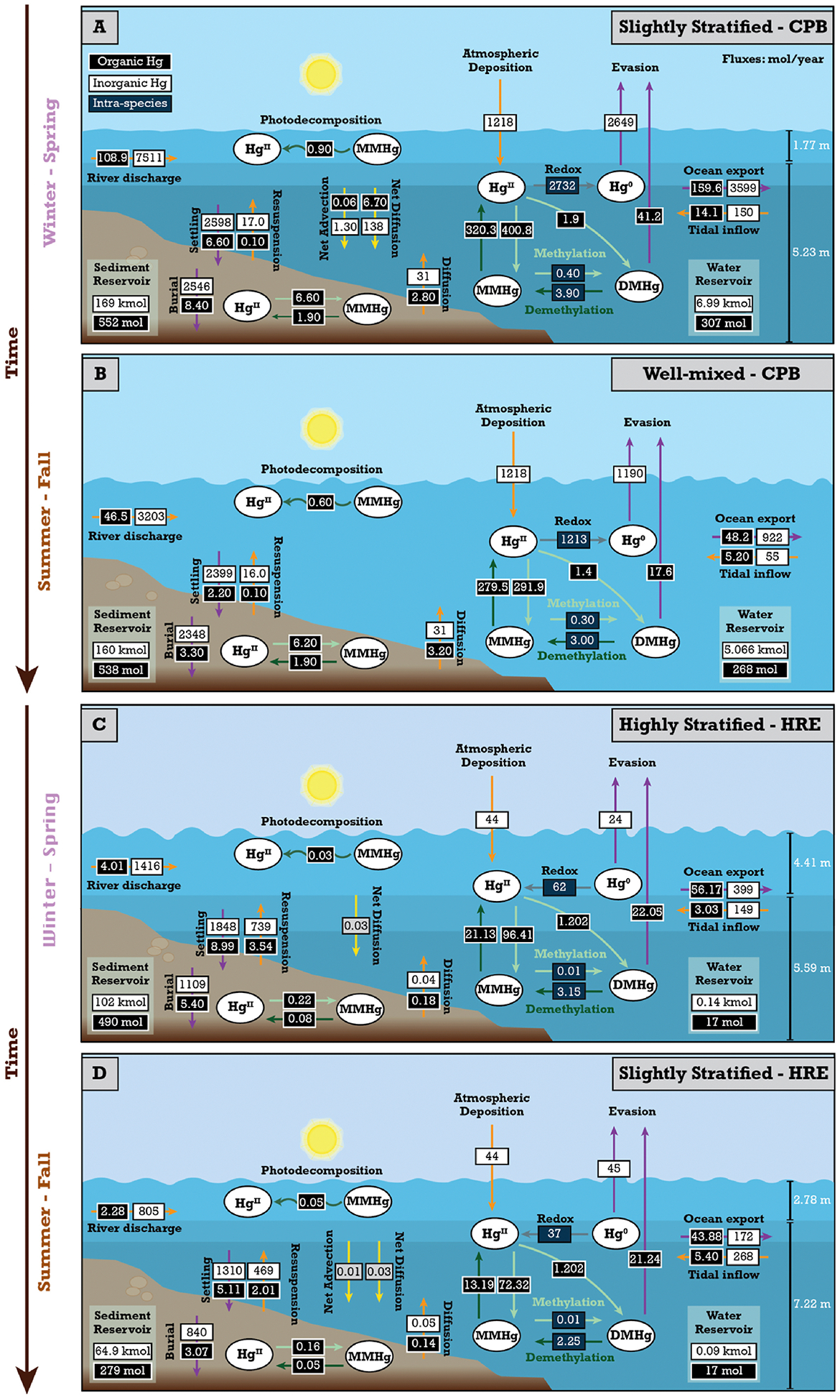
Mass budget of mercury (Hg) cycling in the estuaries under different stratification conditions at steady state. A) Chesapeake Bay (CPB) under slightly stratified conditions during periods of increased river discharge. B) Chesapeake Bay (CPB) under well-mixed conditions during periods of decreased river discharge. C) Hudson River Estuary (HRE) under highly stratified conditions during periods of increased river discharge. D) Hudson River Estuary (HRE) under slightly stratified conditions during periods of decreased river discharge. The inorganic Hg reservoirs are in kmol, and the organic Hg reservoirs are in mol. Each arrow color highlights a distinct pathway in the Hg cycling process, with orange arrows representing external inputs into the water column, purple arrows representing outputs from the water column, green and gray arrows representing biogeochemical transformations between the four main Hg species, and yellow arrows representing the estuarine mixing mechanisms. Tidal inflow and ocean export are shown separately to illustrate the magnitude of each directional flux, but together represent the bidirectional tidal exchange process.

Fig. 5A and B shows that CPB transitions from well-mixed to slightly stratified conditions when river discharge increases, resulting in a 20% increase in THg export to the coastal ocean. This is because river discharge accounts for 71–85% of the annual THg input to CPB, while tidewater inflow and atmospheric deposition contribute less than 30% of the annual THg input. The fraction of THg removed through burial in sediment also varies with stratification, with well-mixed conditions leading to 25% more THg being buried in estuarine sediments compared to slightly stratified conditions. This is again due to increased residence time in well-mixed conditions, which enhances sedimentation efficiency for particle-bound Hg and improves sediment mixing from wave action and tidal forces. Our model’s sensitivity to particle settling indicates that these processes effectively lower Hg concentrations in the water column and enhance its burial in estuarine sediment. This aligns with findings in other coastal environments, such as the Gulf of Trieste, where Hg concentrations in the settling sediment particles were found to be of the same order of magnitude as the amount of Hg observed in the surface sediments (Pavoni et al., 2023), further showing that settling processes play a crucial role in the transfer of particle-bound Hg from the water column to the sediment. The evasion flux in CPB is highest under slightly stratified conditions due to a larger pool of Hg^0^ and DMHg in the surface water, higher wind speeds, and an increased reduction of Hg^II^ to Hg^0^ (Fig. 5A). Slight stratification allows for the input of Hg^0^ and DMHg from depth to advect to the surface, where it can easily evade into the atmosphere under suitable conditions. This advective transport process is absent in well-mixed conditions due to the uniform salinity of the water column. In addition, tidal circulation influences the vertical and horizontal distribution of this Hg species in the slightly stratified systems, with diffusive and advective transport processes redistributing Hg throughout the water column, allowing for more frequent exchanges between the surface and bottom water layers (Huguenard et al., 2015; Zou et al., 2016).

In contrast, we see that HRE transitions from slightly stratified to highly stratified conditions as river discharge increases (Fig. 5C and D), resulting in a 9% increase in THg export to the coastal ocean. Like CPB, river discharge constitutes 71–88% of the annual THg input to HRE, while tidewater inflow and atmospheric deposition account for less than 30 % of the annual THg input. The fraction of THg removed through burial in sediment is also influenced by stratification, with slightly stratified conditions leading to 6% more THg being buried compared to highly stratified conditions. This variation arises from the strong pycnocline in highly stratified conditions. This pycnocline limits vertical mixing, causing Hg species that settle to the bottom layer to remain trapped in the bottom layer, extending their residence time and enhancing particle-bound Hg deposition (Dellapenna et al., 2020). The evasion flux of gaseous Hg in HRE mirrors that of CPB, with the highest flux observed under slightly stratified conditions due to the accumulation and subsequent release of Hg^0^ and DMHg from the surface waters (Fig. 5D). Observations from Long Island Sound (Rolfhus and Fitzgerald, 2001), an estuary 28 km from HRE, further support these findings, as higher concentrations of dissolved gaseous Hg and saturation levels were recorded in the surface waters of Long Island Sound during summer when HRE was also slightly stratified. These further show that the local hydrodynamics and climatic conditions that influence stratification in HRE contribute to higher evasion of Hg^0^ and DMHg during periods of slight stratification.

Our findings highlight the important role that river discharge plays in controlling Hg input and stratification dynamics, which in turn influences Hg export and other removal processes in estuarine systems. In a changing climate, increasing storm runoff and freshwater input into estuaries are expected to enhance stratification, thereby increasing Hg export to the coastal ocean. Land use changes, such as deforestation, can further exacerbate this process by remobilizing previously deposited Hg in the terrestrial environment globally (170–300 Mg yr^−1^) (Lacerda et al., 2004; Obrist et al., 2018). It is estimated that 1088 ± 379 Gg of Hg is stored in the global surface soil (Wang et al., 2019), and land use changes can remobilize this stored Hg and result in elevated concentrations in river discharge. Moreover, estuaries deliver significant amounts of nutrients and organic matter to the coastal ocean (Cloern, 2001), which can stimulate biological activity (Cloern et al., 2014) and MeHg formation, potentially contributing to higher Hg burdens in coastal communities. Our model demonstrates that while well-mixed conditions in CPB act as a substantial sink for riverine Hg, highly and slightly stratified conditions in both estuaries enhance Hg export to the coastal ocean, potentially elevating coastal Hg concentrations and posing risks to marine ecosystems and human health.

### 3.3. The presence of stratification in the water column enhances the production and export of estuarine MeHg to the coastal ocean

Similarly, as in the previous section, we use the model to investigate how seasonal changes in the strength of stratification affect the production of MeHg in CPB and HRE. Additionally, we examine how these changes influence the quantity of MeHg exported to the coastal ocean from the two estuaries.

**Fig. 6.**
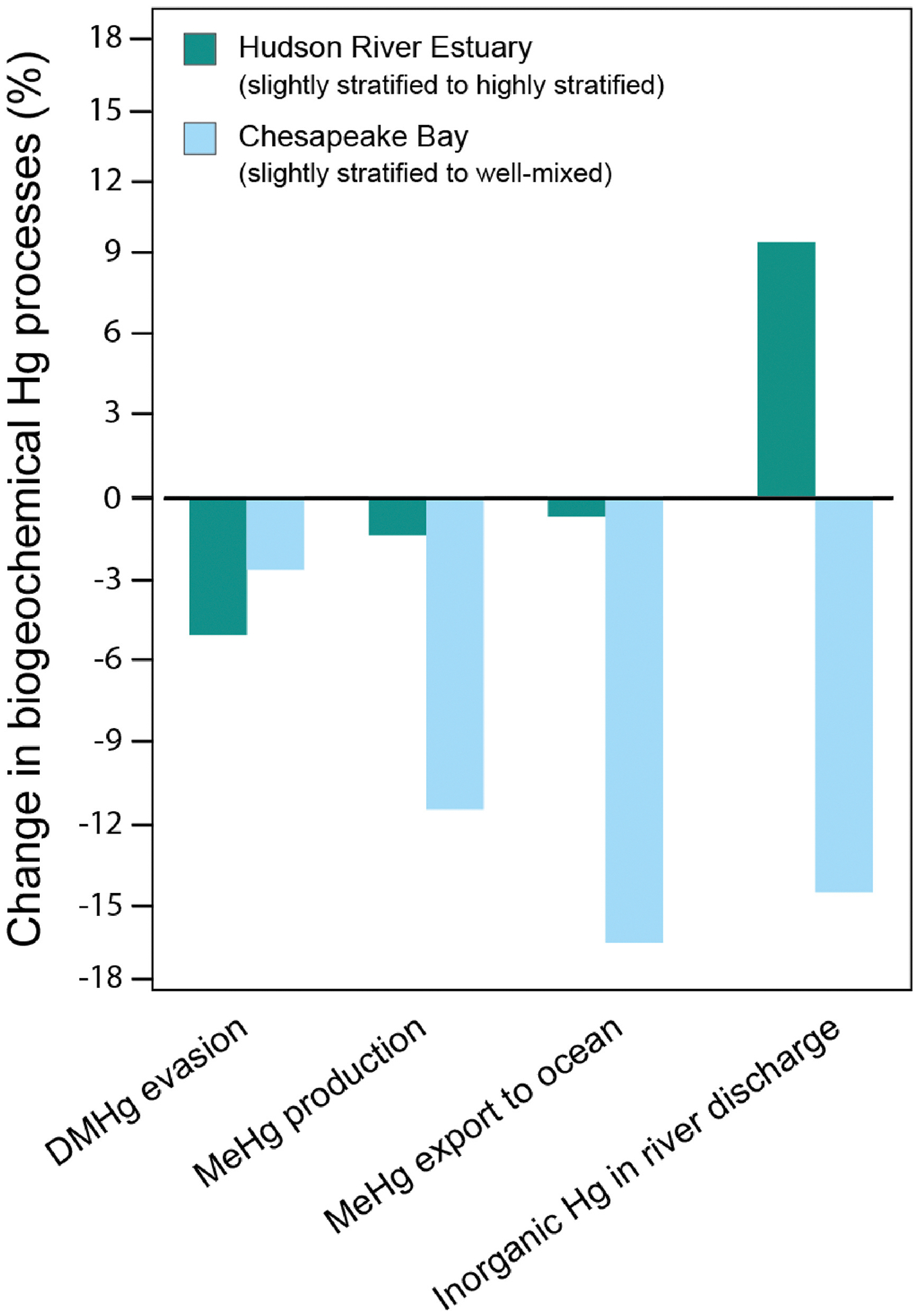
Changes in biogeochemical mercury (Hg) processes under different estuary stratification conditions. The bar chart illustrates the variations in methylmercury (MeHg) production, dimethylmercury (DMHg) evasion, the export of MeHg to the coastal ocean, and the input of riverine inorganic Hg between the Hudson River Estuary (shown in dark green) and Chesapeake Bay (shown in light blue) as the systems transition from slightly stratified to other stratification conditions (well-mixed and highly stratified).

In CPB, as the system transitions from slightly stratified to well-mixed conditions, the MeHg production decreases by 11.5%, leading to a 16.4% decrease in the quantity of MeHg exported to the coastal ocean annually (Fig. 6). We attribute the higher MeHg production under slightly stratified conditions to greater river discharge (Fig. 3A), which delivers 14.5% more inorganic Hg to the slightly stratified system (Figs. 5A–6). This higher river influx increases the bioavailable pool of dissolved Hg^II^, the primary substrate for MeHg formation (Bravo and Cosio, 2020; Munson et al., 2018), as stratification intensifies within the water column. This is consistent with findings from Mason et al. (2012), which highlight the importance of riverine Hg loading in controlling MeHg concentrations in estuarine and coastal environments. Moreover, the increase in net primary production under slightly stratified conditions favors microbial Hg^II^ methylation, thereby facilitating MeHg production (Mason et al., 1999). Earlier studies have also shown that increased primary production boosts the availability of organic matter (Eckley et al., 2021), which, when decomposed, consumes oxygen and contributes to the formation of anoxic zones, thus promoting the methylation of Hg^II^.

In the HRE, the transition from slightly stratified to highly stratified conditions leads to a 1.6% decrease in MeHg production. This shift results in a 0.7% decrease in the amount of MeHg exported to the coastal ocean annually (Fig. 6). The decrease in MeHg production in the highly stratified conditions, despite a 9.4% increase in inorganic Hg input from river discharge, can be attributed to the shorter residence time of the water in the surface mixed layer. This leads to a higher flushing rate (Marsooli et al., 2018; Lemagie and Lerczak, 2014; Zhang et al., 2010), resulting in less time for Hg^II^ to undergo methylation within the estuary. The increase in MeHg export to the coastal ocean under slightly stratified conditions is also attributed to the advective and diffusive mixing between the surface mixed layer and the stratified bottom layers. This mixing allows some of the MeHg produced at greater depths to reach the surface (Adams et al., 2024), where it can be readily exported to the coastal ocean. In contrast, during highly stratified conditions, strong pycnoclines restrict the mixing of MeHg produced in the bottom layers, preventing it from reaching the surface. As a result, MeHg accumulates in the stratified bottom layers, where it undergoes further demethylation and a portion of it is eventually deposited into the sediment (Fig. 5C) (Mason et al., 1999). This means that when stratification is high, there is less MeHg available in the highly productive surface waters, which may lead to less biological uptake depending on the depth of the euphotic zone, the location of phytoplankton, and other estuarine mixing processes, possibly resulting in lower MeHg accumulation in organisms at the base of the food chain. Despite this, MeHg accumulation in the more stratified bottom layer remains available to deep-dwelling organisms.

Our study highlights the dual role of estuaries in both exporting and retaining Hg, as well as the role of stratification and estuarine mixing in the formation of MeHg from Hg species entering estuaries via river discharge. Studies show that many estuaries may experience enhanced water column stratification in the coming decades due to climate change. While exact figures have not yet been published, modeling studies using regional conditions and specific climate scenarios suggest that estuaries—particularly those in temperate regions—could exhibit increased stratification as a result of rising sea levels, warming surface waters, reduced wind mixing, and altered freshwater inflows. Increased freshwater input into estuaries is likely to enhance stratification due to the difference in density between freshwater (less dense) and seawater (more dense) (Shen et al., 2022; Duvall et al., 2022; Ni et al., 2019); this density gradient (pycnocline) separates the less dense freshwater in the surface layer and the denser, saline water below (Shen et al., 2022; Geyer, 2010; Geyer and Ralston, 2011b). This strong pycnocline weakens vertical oxygen exchange and can lead to the development of near-bed hypoxia (Hong and Shen, 2012; Duvall et al., 2022; Pein et al., 2021), a condition known to favor the formation of MeHg (Mason et al., 1999; Bravo and Cosio, 2020). Our findings reveal that MeHg production and export to the coastal ocean increased by 11.5% and 16.4%, respectively, when the stratification conditions in CPB shifted from well-mixed to slightly stratified. This indicates that many estuaries may experience an increased export of MeHg to the coastal ocean as the climate changes. Future work should consider how changes in estuary stratification, land use, and hydrology affect not only Hg export to the ocean but also long-term retention and transformation within the estuarine environment.

## Supplementary Material

supplementary data

## Figures and Tables

**Table 1:** Chesapeake Bay and Hudson River Estuary range (geometric mean; geometric standard deviation) of mercury (Hg) species concentrations, including inorganic (Hg^II^), elemental (Hg^0^), and methylmercury (MeHg) in the water column, porewater, and sediment from the literature. These values were used to establish reservoir sizes and run the box model.

	Hg species	Chesapeake Bay	Hudson River Estuary
Water (pM)	Hg^II^	5–20 (**10.00; 1.41**)^a^	87–581 (**224.83; 1.61**)^c^
	Hg^0^	0.1–0.25 (**0.16; 1.26**)^a^	4.35–29 (**11.23; 1.61**)^c^
	MeHg	0.02–1 (**0.14; 2.66**)^a^	0.22–0.65 (**0.38; 1.31**)^c^
Porewater (pM)	Hg^II^	5–23.6 (**10.86; 1.47**)^b^	2.2–78.4 (**13.13; 2.44**)^c^
	MeHg	0.24–2.4 (**0.76; 1.78**)^b^	0–1.8 (**0.89; 2.99**)^c^
Sediment (pmol/g)	Hg^II^	100–850 (**291.55; 1.71**)^b^	3000–9000 (**5196; 1.32**)^c^
	MeHg	1–5 (**2.24; 1.50**)^b^	3.1–12.5 (**6.22; 1.42**)^c^

a Measurements in the surface waters of the Chesapeake Bay system (Mason et al., 1999).

b Bottom sediment measurement in the mainstem of the Chesapeake Bay and the mid-Atlantic continental margins during four cruises: May 2005, July 2005, August–September 2005, and April 2006 (Hollweg et al., 2009).

c Measurement in the estuarine turbidity maximum of the HRE between October 2000 and June 2001 (Heyes et al., 2004).

## Data Availability

The data used is the body of the texts and supplementary information.

## Appendix A. Supplementary data

Supplementary data to this article can be found online at https://doi.org/10.1016/j.ecss.2025.109595.
