# Supplementary material for: Salinity-driven stratification enhances riverine mercury export to the coastal ocean
Source: Estuar Coast Shelf Sci. Author manuscript; Available in PMC 2025 Dec 17. (PMC12707809; doi:10.1016/j.ecss.2025.109595)
Supplement: supplementary data [file NIHMS2128152-supplement-supplementary_data.docx]

Supplemental Information

Salinity-Driven Stratification Enhances Riverine Mercury Export to the Coastal Ocean

Roland P. Ovbiebo^a*^, Cathryn D. Sephus^a^, Amina T. Schartup^a*^

^a^Scripps Institution of Oceanography, University of California San Diego, USA.

Corresponding author: Roland Ovbiebo, [rovbiebo@ucsd.edu](mailto:rovbiebo@ucsd.edu) and Amina T. Schartup, aschartup@ucsd.edu

Summary: 26 pages, 2 figures, 16 tables, 31 equations, and 2 texts

Table of Contents

[Fig. S1. Map of the study area showing the location of Chesapeake Bay (left) and Hudson River Estuary (right) in the United States. 2](#_Toc199232345)

[Table S1: Hg species in Chesapeake Bay water column and sediment used to run the box model. 3](#_Toc199232346)

[Table S2: Hg species in Hudson River Estuary water column and sediment used to run the box model. 4](#_Toc199232347)

[Table S3: External Hg inputs. 5](#_Toc199232348)

[Table S4: Physical characteristics used to develop mass budgets for Hg speciation in the estuary. 6](#_Toc199232349)

[Table S5: Hydrological Budget 7](#_Toc199232350)

[Table S6: Residence time calculation using the freshwater method [36–38]. 8](#_Toc199232351)

[Text 1: Hg transport processes between the reservoirs 8](#_Toc199232352)

[Table S7: Vertical advection transfer in the water column. 9](#_Toc199232353)

[Table S8: Solids budget. 10](#_Toc199232354)

[Table S9: Diffusion in sediment and water column following the methods of Sunderland et al. [43] and Soerensen et al. [41]. 11](#_Toc199232355)

[Table S10: Settling to subsurface and sediment reservoir. 13](#_Toc199232356)

[Table S12: Hg chemical transformation in the sediment. 15](#_Toc199232357)

[Text 2: Hg biogeochemical transformation 15](#_Toc199232358)

[Table S13: Gas exchange of DMHg based on Nightingale et al. [65] calculations. 17](#_Toc199232359)

[Table S15: Shortwave radiation in the water column. 19](#_Toc199232360)

[Table S16: Hg chemical transformation in the water column. 20](#_Toc199232361)

[Table S16: Hg chemical transformation in the water column (Continued). 21](#_Toc199232362)

[Fig. S2. Variation in the amount of (A) THg and (B) MeHg exported to the ocean due to ±15% change in key Hg biogeochemical transformation parameters in Chesapeake Bay. 21](#_Toc199232363)

[References 22](#_Toc199232364)


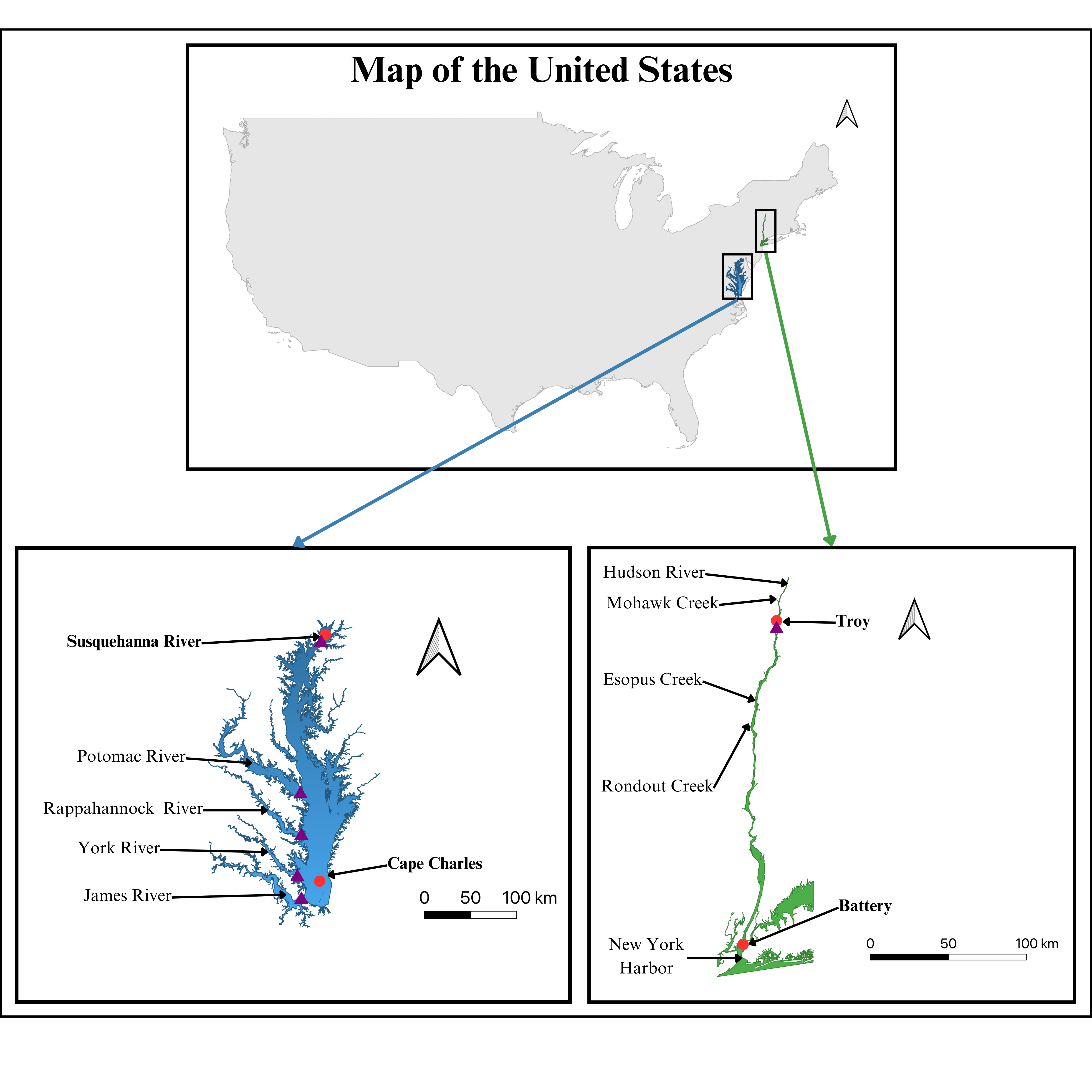


**Fig. S1.** Map of the study area showing the location of Chesapeake Bay (left) and Hudson River Estuary (right) in the United States. The red circles show the length of the two estuaries. The map in the upper panel shows the locations of both estuaries in the continental United States. The lower-left panel provides a detailed view of the Chesapeake Bay, and the lower-right panel shows the Hudson River Estuary. The red circles indicate the approximate lengths of each estuary, while the purple triangles represent their respective river mouths. Scale bars in each estuary panel give approximate distances.

# **Table S1:** Hg species in Chesapeake Bay water column and sediment used to run the box model.

| Parameter | | Initial Concentration  (pM) | | Dissolved Reservoir Size  (mol) | | Particulate Reservoir Size  (mol) | Reference |
| --- | --- | --- | --- | --- | --- | --- | --- |
| ***Surface Mixed Layer (MSL)***  ^+^*MMHg_dis_so_* \| *MMHg_part_so_* | | | 0.084 | 1.60 | 0.05 | | Ref. [1] |
| *Hg^0^_so_* | | | 0.16^a^ | 3.07 |  | | Ref. [1] |
| *^*^Hg^II^_dis_so_* \| *Hg^II^_part_so_* | | 10.00 | | 159 | 35.29 | | Ref. [1] |
| ^+^*DMHg_so_* | | 0.057 | | 1.10 |  | | Ref. [1] |
| ***Stratified Bottom Layer (SBL)*** | | | | |  | |  |
| ^+^*MMHg_dis_ss_* \| *MMHg_part_ss_* | | 0.084 | | 4.98 | 0.27 | | Ref. [1] |
| *Hg^0^_ss_* | | 0.16^a^ | | 9.77 |  | | Ref. [1] |
| *^*^Hg^II^_dis_ss_* \| *Hg^II^_part_ss_* | | 10.00 | | 432.70 | 185.16 | | Ref. [1] |
| ^+^*DMHg_ss_* | | 0.057 | | 3.50 |  | | Ref. [1] |
| ***Sediment porewater*** | | | | |  | |  |
| *MMHg_pw_* | 0.76 | | |  |  | | Ref. [2] |
| *^#^Hg^II^_pw_*  ***Sediment*** | | 10.86 | |  |  | | Ref. [2] |
| *MMHg_sed__dis* \| *MMHg_sed_p_*  ^#^*Hg^II^_sed_dis_* \| *Hg^II^_sed_p_* | | 2.23  291.55 | | 0.33  4.28 | 695  90660 | | Ref. [2]  Ref. [2] |

The reservoir size was calculated by the Hg species’ initial concentration in the water column and sediment.

^+^ Based on average ratios from previous studies, we assumed that the concentration of MMHg is 60% and DMHg is 40% of methylmercury (MeHg), respectively [3–6].

*^*^*The Hg^II^ in the water column was calculated as the difference between the THg and the sum of Hg^0^ and MeHg.

*^a^*The Hg^0^ concentration is assumed to be about the same as that of dissolved gaseous mercury (DGM) since it is estimated that DGM contains more than 90% of Hg^0^ in surface water [7].

*^#^*The Hg^II^ in the sediment and porewater was calculated as the difference between the THg and MeHg.

# **Table S2:** Hg species in Hudson River Estuary water column and sediment used to run the box model.

| Parameter | Initial Concentration  (pM) | | | | Dissolved Reservoir Size  (mol) | Particulate Reservoir Size  (mol) | Reference |
| --- | --- | --- | --- | --- | --- | --- | --- |
| ***Mixed Surface Layer (MSL)***  ^+^*MMHg_dis_so_* \| *MMHg_part_so_* | | | | 0.23 | 0.275 | 0.072 | Ref. [8] |
| *Hg^0^_so_* | | | | 11.23^a^ | 17.20 |  | Ref. [8] |
| *^*^Hg^II^_dis_so_* \| *Hg^II^_part_so_* | | 224.83 | | | 17.64 | 326.60 | Ref. [8] |
| ^+^*DMHg_so_* | | 0.15 | | | 0.232 |  | Ref. [8] |
| ***Stratified Bottom Layer (SBL)*** | | | | | |  |  |
| ^+^*MMHg_dis_ss_* \| *MMHg_part_ss_* | | 0.23 | | | 0.280 | 0.21 | Ref. [8] |
| *Hg^0^_ss_* | | 11.23^a^ | | | 24.29 |  | Ref. [8] |
| *^*^Hg^II^_dis_ss_* \| *Hg^II^_part_ss_* | | 224.83 | | | 8.99 | 477.16 | Ref. [8] |
| ^+^*DMHg_ss_* | | 0.15 | | | 0.327 |  | Ref. [8] |
| ***Sediment porewater*** | | | | | |  |  |
| *MMHg_pw_* | | 0.89 | | |  |  | Ref. [8] |
| *^#^Hg^II^_pw_*  ***Sediment*** | | | 13.13 | |  |  | Ref. [8] |
| *MMHg_sed__dis* \| *MMHg_sed_p_*  ^#^*Hg^II^_sed_dis_* \| *Hg^II^_sed_p_* | | | 6.22  5196.15 | | 0.001  0.012 | 30.81  25716.82 | Ref. [8]  Ref. [8] |

The reservoir size was calculated by the Hg species’ initial concentration in the water column and sediment.

^+^ Based on average ratios from previous studies, we assumed that the concentration of MMHg is 60% and DMHg is 40% of methylmercury (MeHg), respectively [3–6].

*^*^*The Hg^II^ in the water column was calculated as the difference between the THg and the sum of Hg^0^ and MeHg.

^a^The concentration of Hg^0^ in the water column is assumed to be 5% of THg concentrations based on the typical average percentage of THg concentration in various aquatic environments [1,9–11].

*^#^*The Hg^II^ in the sediment and porewater was calculated as the difference between the THg and MeHg.

# **Table S3:** External Hg inputs.

| Parameter | Units | | | Description | Chesapeake Bay | | | | | Hudson River Estuary | | | Reference | |
| --- | --- | --- | --- | --- | --- | --- | --- | --- | --- | --- | --- | --- | --- | --- |
| ***River discharge***  *^+^MMHg_riv_* | pM | | | MMHg concentration in river inflow | | 0.66 | | | | 0.13 | | Ref. [1,12] | | |
| *Hg^0^_riv_* | pM | | | Hg^0^ concentration in river inflow | | 0.25^a^ | | | | 3.61^b^ | | Ref. [1,12] | | |
| *Hg^II^_riv_* | pM | | | Hg^II^ concentration in river inflow | | 75.6 | | | | 72 | | Ref. [12,13] | | |
| *^+^DMHg_riv_* | pM | | | DMHg concentration in river inflow | | 0.44 | | | | 0.09 | | Ref. [12,13] | | |
| ***Tidal inflow*** | |  | | | | | | | | |  | | | |
| *^+^MMHg_tf_* | pM | | | MMHg concentration in tidal inflow | | | 0.06 | | 0.15 | | | | | Ref. [14,15] |
| *Hg^0^_tf_* | pM | | | Hg^0^ concentration in tidal inflow | | | 0.05 | | 0.19 | | | | | Ref. [14,15] |
| *Hg^II^_tf_* | pM | | | Hg^II^ concentration in tidal inflow | | | 0.8 | | 2.54 | | | | | Ref. [14,15] |
| *^+^DMHg_tf_* | pM | | | DMHg concentration in tidal inflow | | | 0.02 | | 0.1 | | | | | Ref. [14,15] |
| ***Atmospheric deposition*** | | |  | | | | | | | |  | | | |
| *MMHg_wet_* | nmol m^-2^ yr^-1^ | | | MMHg average wet deposition | | | 0.46^c^ |  | | | Ref. [16] | | | |
| *Hg^II^_wet_* | nmol m^-2^ yr^-1^ | | | Hg^II^ average wet deposition | | | 92 |  | | | | | | Ref. [16] |
| *Hg_dep_* | nmol m^-2^ yr^-1^ | | | Total mercury annual deposition | | | 106 | 119^d^ | | | | | | Ref. [16,17] |

^+^Based on average ratios from previous studies, we assumed that the concentration of MMHg is 60% and DMHg is 40% of methylmercury (MeHg), respectively [3–6].

*^a^*The Hg^0^ concentration is assumed to be about the same concentration of dissolved gaseous mercury (DGM) since it is estimated that DGM contains >90% of Hg^0^ in surface water [7].

^b^The concentration of Hg^0^ in the water column is assumed to be 5% of THg concentrations based on the typical average percentage of THg concentration in various aquatic environments [3,4,18–20].

^c^The concentration of MMHg in rain is estimated to be about 0.5% of the THg [21].

^d^No individual Hg species wet deposition data were available for the Hudson River Estuary, so we used the bulk Hg deposited.

# **Table S4:** Physical characteristics used to develop mass budgets for Hg speciation in the estuary.

| Parameter | Units | Description | Chesapeake Bay | Hudson River Estuary | Reference |
| --- | --- | --- | --- | --- | --- |
| $T_{w\_ss}$ | °C | Average water temperature of the SBL | 14.57 | 13.38 | Ref. [22,23] |
| ${Sal\_}_{so}$ | psu | Average salinity of the MSL | 13.73 | 14.97 | Ref. [22,23] |
| ${Sal\_}_{ss}$ | psu | Average salinity of the SBL | 15.58 | 21.57 | Ref. [22,23] |
| ${Sur\_}_{sal\_oc}$ | psu | Average ocean salinity into the MSL | 25.1 | 25.49 | Ref. [23,24] |
| ${Sub\_}_{sal\_oc}$ | psu | Average ocean salinity into the SBL | 27.1 | 28.85 | Ref. [23,24] |
| $S_{dep}$ | m | Depth of the stratified bottom layer | Equation 2 | |  |
| $W_{dep}$  $M_{dep}$ | m  m | Average water depth  Depth of the mixed mixed layer | 7  1.5-2.1 | 10  1.6-6.1 | Ref. [25,26]  This study |
| *^#^ρ* | g cm^-3^ | Average water density | 1.0104 | 1.0131 | This study |

^#^The water density of the system was calculated using the Python package Gibbs SeaWater (GSW) Oceanographic Toolbox of TEOS-10.

# **Table S5:** Hydrological Budget

| Parameter | Units | Description | Chesapeake Bay | Hudson River Estuary | Reference |
| --- | --- | --- | --- | --- | --- |
| ${E\_}_{len}$ | m | Length of the box | 3 x 10^5^ | 2.5 x 10^5^ | Ref. [27] |
| ${E\_}_{width}$ | m | Width of the box | 3.9 x 10^4^ | 1.5 x 10^3^ |  |
| *^#^SAw/SAs* | m^2^ | Surface area of the estuary/sediment | ${E\_}_{len}$ * ${E\_}_{width}$ | |  |
| tanθ | unitless | Shore gradient | 1 x 10^-4^ - 1 x 10^-3^ | 1 x 10^-3^ - 5 x 10^-2^ | Ref. [28–30] |
| Δh | m | Tidal range | 0.3-1 | 1-1.5 | Ref. [31–33] |
| ${I\_}_{width}$ | m | Intertidal width | $\frac{\Delta h}{tan\theta}$ | |  |
| *SAt* | m^2^ | Intertidal surface area | ${E\_}_{len}$ * ${I\_}_{width}$ | |  |
| *Vol_w_so_* | m^3^ | Volume of the MSL | *SAw ** $W_{dp\_so}$ | |  |
| *Vol_w_ss_* | m^3^ | Volume of the SBL | *SAw ** $S_{dep}$ | |  |
| *V_sed_* | m^3^ | Volume of the sediment | *SAs* * *Sed_z* | |  |
| $P_{rate}$ | m s^-1^ | Average precipitation rate | 0.7-1.4 x 10^-1^ | 0.4-1.5 x 10^-2^ | Ref. [34,35] |
| $Pre$ | m^3^ s^-1^ | Precipitation inflow into the estuary | $P_{rate}$ * *SAw* | |  |
| *^&^*${Riv}_{fl}$ | m^3^ s^-1^ | River inflow into the estuary | 971-4064 | 226-862 | Ref. [36–38] |
| ${outflow}_{\_so}$ | m^3^ yr^-1^ | Outflow from the MSL | $\left( \frac{Vw_{so}}{{Flu}_{so} * 365.25} \right)$ | |  |
| ${outflow}_{\_ss}$ | m^3^ yr^-1^ | Outflow from the SBL | $\left( \frac{Vw_{ss}}{{Flu}_{ss} * 365.25} \right)$ | |  |
| ${Tinflo}_{\_so}$ | m^3^ yr^-1^ | Tidal inflow into the MSL | ${outflow}_{\_so}- Pre$ $-{Riv}_{fl}$ | |  |
| ${Tinflo}_{\_ss}$ | m^3^ yr^-1^ | Tidal inflow into the SBL | ${outflow}_{\_so}+ Pre$ $+{Riv}_{fl}$ $-$ ${outflow}_{\_so}$ | |  |
| *Sed_z* | m | Depth of active bottom sediment | 0.04 | 0.02 | Ref. [39,40] |

^#^The surface area of the water column is assumed to be the same for the sediment.

^&^The daily river discharge was obtained from the U.S. Geology Survey (USGS), where the long-term seasonal average river discharge was calculated.

# **Table S6:** Residence time calculation using the freshwater method .

| Parameter | Units | Description | Value |
| --- | --- | --- | --- |
| ${Flu}_{so}$ | day | Average flushing time in the MSL | (*Vol_w_so_* * ${FWF}_{so}$) / ${FW}_{fl}$* (60 * 60 * 24) |
| ${Flu}_{ss}$ | day | Average flushing time of the SBL | (*Vol_w_ss_* * ${FWF}_{ss}$) / ${FW}_{fl}$* (60 * 60 * 24) |
| ${FWF}_{so}$ | unitless | Freshwater fraction of the MSL | $\frac{{(Sur\_}_{sal\_oc} - {Sal}_{\_so} )}{{Sur\_}_{sal\_oc}}$ |
| ${FWF}_{ss}$ | unitless | Freshwater fraction of the SBL | $\frac{{(Sub\_}_{sal\_oc} - {Sal}_{\_ss} )}{{Sub\_}_{sal\_oc}}$ |
| ${FW}_{fl}$ | m^3^ s^-1^ | Freshwater inflow rate | ${Riv}_{fl}$ + $Pre$ |

# **Text S1:** Hg transport processes between the reservoirs

This section describes how we calculated the flux of the different hydrodynamic processes exchanging Hg species between each reservoir, as shown in Figure 1 of the main manuscript.

*Advection:* Advective transport of Hg species between water column compartments was based on the system length, depth-average vertical current velocity, and measured Hg concentrations in each reservoir (**Table S7**). Because vertical mixing is suppressed by stratification [44], we only consider vertical advective mixing in the slightly stratified systems.

*Diffusion:* The diffusion flux of Hg^II^ and MMHg from the sediment porewater to the overlaying water is calculated based on Fick’s law, taking into account the seabed's properties, including the temperature-corrected diffusion coefficient, tortuosity, porosity of the sediment, and depth of the active sediment layer (**Table S8-9**) [45]. In the water column, the eddy diffusion flux was based on concentration gradients between the base of the surface mixed layer, the average mid-depth peak, the concentration of Hg species, and the vertical eddy diffusivity coefficient (**Table S9**) [46]**.**

*Settling:* The model combines the physical and hydrodynamic properties of the particles and systems to estimate the suspended sediment settling velocity based on Stokes’s law to account for the influencing factors of settling velocity, such as particle size, shape, and density (**Table S10**) [47]. The fraction of dissolved Hg^II^ and MMHg in the water column was calculated from the particle-water partition coefficient and the suspended solids concentration in the water column (**Table S10**) [48]. The settling flux of particulates Hg^II^ and MMHg in the water columns and benthic sediments was then calculated as the product of the fraction of Hg^II^ and MMHg in the solid phase concentration and particle settling velocity (**Table S10**). The net fluxes of particulates Hg across the sediment-water interface are estimated as the difference between settling and resuspension, which co-occur [49].

*Resuspension****:*** The erosion rate, erosion rate parameter, combined wave-plus-current-induced bottom shear stress, critical shear stress of erosion, system surface area, the volume of sediment, and the fraction of dissolved Hg^II^ and MMHg in the sediment were used to calculate the resuspension rate (**Table S11**) [50,51]. Resuspension occurs in the model when and where bottom shear stress exceeds critical shear stress, and the erosion rate varies depending on hydrodynamic conditions and the local sediment grain size distribution [49].

*Burial:* Burial occurs in the model when Hg is bound to particles and settles out of the water column. In the active surficial layer of the sediment, both Hg^II^ and MMHg are continually transported down to deeper layers, which are treated as a permanent sink within the model (**Fig. 1**). This process highlights the dynamic movement of Hg in sediment and its eventual burial, contributing to the long-term storage and potential transformation of Hg compounds in aquatic systems. The total burial flux of Hg^II^ and MMHg in the sediment is the sum of burial in intertidal areas and the main estuary based on the solids budget and Hg concentration in the sediment (**Table S12**). We divided this total flux by the surface area of the sediments to calculate an average basin-wide burial rate [48]. Rate coefficients for sediment burial flux of Hg^II^ and MMHg were calculated from basin-wide burial rate, the benthic and intertidal sediment surface area, the sediment volume, and the fraction of Hg^II^ and MMHg in the dissolved phase of benthic sediments [48].

This equation calculates the advection flux of Hg species in the water column. The other variables are described in Table S7 and previous tables:

$A_{flux}$ = [$\frac{{vel}_{\_u} * C_{w}X}{{E\_}_{len}}$] ** (60 * 60 * 24)* [Equation S1]

$M_{adv}$ = $A_{flux}$*** $SAw$ * 1 x 10^-12^ [Equation S2]

# **Table S7:** Vertical advection transfer in the water column.

| Parameter | Units | Description | Chesapeake Bay | Hudson River Estuary | Reference |
| --- | --- | --- | --- | --- | --- |
| $M_{adv}$ | mol day^-1^ | Water column net advection flux of Hg species in the two systems | Equation S1 | |  |
| $A_{flux}$ | pmol m^-2^ day^-1^ | Advective flux of X species (Hg^II^, Hg^0^, MMHg, and DMHg) in water column | Equation S2 | |  |
| ${vel}_{\_u}$ | m s^-1^ | Averaged vertical current velocity | 0.13 | 0.48-0.87 | Ref. [23,52] |
| $C_{w}X$ | pM | Concentration of X species (Hg^II^, Hg^0^, MMHg, and DMHg) in water column | Table S1 | Table S2 |  |

# **Table S8:** Solids budget.

| Parameter | Units | Description | Chesapeake Bay | Hudson River Estuary | Reference |
| --- | --- | --- | --- | --- | --- |
| *NPP* | gC m^-2^ day^-1^ | Average Net Primary Productivity | 1.5 | 0.19 | Ref. [53,54] |
| *SPM_so* | kg L^-1^ | Concentration of particles in MSL | 8.4 x 10^-6^ | 1.5-2.4 x 10^-5^ | Ref. [23,55] |
| *SPM_ss* | kg L^-1^ | Concentration of particles in SBL | 1.7 x 10^-5^ | 3.2-6.8 x 10^-5^ | Ref. [23,55] |
| *sedsolids* | kg L^-1^ | Concentration of solids in the benthic sediment | 0.67 | | Ref. [11] |
| $B_{rate\_mb}$ | m yr^-1^ | Mean burial rate of benthic sediment solids | 0.006 | 0.002 | Ref. [56,57] |
| $B_{rate\_it}$ | m yr^-1^ | Mean intertidal burial rate | 0.01 | 0.009 | Ref. [40,58] |

The equations used to calculate the diffusion flux in sediment and water column. The other variables are described in Table S9 and previous tables:

*M_diff_sed_* = ${(D}_{flux\_sed}$* SAs * 1 x 10^-12^) * (60 * 60 * 24) [Equation S3]

$D_{flux\_sed}$ = $\frac{POR* D_{sed\_T}}{TOR}$ * $\frac{\left( C_{w} - C_{pw} \right) * 1000}{D_{max}}$ [Equation S4]

$D_{sed\_T}$ = $\frac{D_{sw25}}{(1+0.048*\left( 25- T_{w_{ss}} \right))}$ [Equation S5]

*M_diff_wa_* = $D_{flux\_wa}$* SAw * 1 x 10^-12^ [Equation S6]

$D_{flux\_wa}$ = $D_{w}$ * ( $\frac{C_{w}X}{D_{ss\_d2}- D_{so\_d1}}$) * (60 * 60 * 24) [Equation S7]

# **Table S9:** Diffusion in sediment and water column following the methods of Sunderland *et al.* [48] and Soerensen *et al.* [46].

| Parameter | Units | | Description | Chesapeake Bay | Hudson River Estuary | Reference |
| --- | --- | --- | --- | --- | --- | --- |
| ***Sediment-water interphase*** | | |  | | |  |
| *M_diff_sed_* | mol day^-1^ | | Sediment-water diffusion flux of Hg^II^ to MMHg | Equation S3 | |  |
| $D_{flux\_sed}$ | pmol m^-2^ day^-1^ | | Diffusive flux based on Fick’s law | Equation S4 | | Ref. [48] |
| *TOR* | unitless | | Tortuosity of sediments | 1 – ln(*POR^2^*) | | Ref. [45] |
| *POR* | unitless | | Porosity of the sediment | 0.9 | 0.73 | Ref. [40,59] |
| $D_{sed\_T}$ | cm^2^ s^-1^ | | Temperature corrected diffusion coefficient | Equation S5 | | Ref. [45] |
| *D_sw25_* | cm^2^ s^-1^ | | Sediment-water diffusion coefficient at 25 °C | 2 x 10^-6^ | | Ref. [45] |
| $D_{max}$ | m | | Depth below surface (m) with maximum porewater Hg concentration | 0.01 | |  |
| $C_{w}$ | pM | | Concentration of Hg^II^ and MMHg in the SBL | Table S1 | Table S2 |  |
| $C_{pw}$ | pM | | Concentration of Hg^II^ and MMHg in the sediment porewater | Table S1 | Table S2 |  |
| ***Water column diffusion*** | |  | | | |  |
| *M_diff_wa_* | mol day^-1^ | | Water column net diffusion flux of Hg species in the two systems | Equation S6 | |  |
| $D_{flux\_wa}$ | pmol m^-2^ day^-1^ | | Diffusion flux of X species (Hg^II^, Hg^0^, MMHg, and DMHg) in water column | Equation S7 | |  |
| $C_{w}X$ | pM | | Concentration of Hg species in the two systems | Table S1 | Table S2 |  |
| $D_{w}$ | cm^2^ s^-1^ | | Eddy diffusion coefficient | 1 x 10^-6^ | 1 x 10^-4^ | Ref. [60,61] |
| $D_{so\_d1}$ | m | | Depth of diffusion gradient in MSL | 2 | |  |
| $D_{ss\_d2}$ | m | | Depth of diffusion gradient in SBL | 4 | |  |

The equations calculate the Hg species' settling flux in the water column. The other variables are described in Table S10 and previous tables:

*k_set_sed_* = $\frac{SAw* s_{vel\_sed}*(1- f_{diss\_ss})}{{Vol}_{w\_ss}}$ [Equation S8]

*k_set_sub_* = $\frac{SAw* s_{vel\_sub}*(1- f_{diss\_so})}{{Vol}_{w\_so}}$ [Equation S9]

*s_vel_sed_* = *[*$\frac{\left( dss - \rho_{ss} \right) *1000 * g * r_{pw}^{2}}{{(f}_{fac} * \mu)}]$ ** (60 * 60 * 24)* [Equation S10]

*s_vel_sub_* = *[*$\frac{\left( dss - \rho_{so} \right) *1000 * g * r_{pw}^{2}}{{(f}_{fac} * \mu)}$*] * (60 * 60 * 24)* [Equation S11]

*f_diss_so_* =$\frac{1}{(1 + {10}^{kp}* SPM\_so)}$ [Equation S12]

*f_diss_ss_*  =$\frac{1}{(1 + {10}^{kp}* SPM\_ss)}$ [Equation S13]

# **Table S10:** Settling to subsurface and sediment reservoir.

| Parameter | Units | Description | Chesapeake Bay | Hudson River Estuary | Reference |
| --- | --- | --- | --- | --- | --- |
| *^#^M_set_sed_* | mol day^-1^ | Settling flux of Hg^II^ and MMHg to the benthic sediment | *Hg^II^_part_ * k_set_sed_ + MMHg_part_ * k_set_sed_* | |  |
| *^#^M_set_sub_* | mol day^-1^ | Settling flux of Hg^II^ and MMHg to the SBL reservoir | *Hg^II^_part_ * k_set_sub_ + MMHg_part_ * k_set_sub_* | |  |
| *k_set_sed_* | day^-1^ | Settling rate of particulate Hg^II^ and MMHg to the benthic sediment | Equation S8 | |  |
| *k_set_sub_* | day^-1^ | Settling rate of particulate Hg^II^ and MMHg to the SBL reservoir | Equation S9 | |  |
| *s_vel_sed_* | m day^-1^ | Settling velocity of suspended solids to the benthic sediment | Equation S10 | |  |
| *s_vel_sub_* | m day^-1^ | Settling velocity of suspended solids to the SBL reservoir | Equation S11 | |  |
| *r_pw_* | m | Average radius of suspended solids | 8.5 x 10^-6^ | 1.5 x 10^-5^ | Ref. [26,62] |
| *g* | m s^-2^ | Gravitational acceleration | 9.81 | |  |
| *^&^μ* | Pa s^-1^ | Dynamic viscosity of seawater | 1.3 x 10^-3^ | 1.1 x 10^-3^ | Ref. [63] |
| *dss* | kg L^-1^ | Density of MSL sediments | 1.5 | | Ref. [48] |
| *f_fac_* | unitless | Friction factor of a sphere | 18 | |  |
| *f_diss_so_* | unitless | Fraction of dissolved Hg^II^ and MMHg in the MSL | Equation S12 | |  |
| *f_diss_ss_* | unitless | Fraction of dissolved Hg^II^ and MMHg in the SBL reservoir | Equation S13 | |  |
| *kp* | L kg^-1^ | Suspended particles partition coefficients for Hg^II^ and MMHg | 5.2 and 4.3 | 5.9 and 4.1 | Ref. [14,64] |

^#^The Hg^II^ and MMHg bound to particles were used to calculate the settling flux in the MSL and SBL systems.

^&^The dynamic viscosity is estimated using the salinity and temperature of the two water reservoirs from Unisense data table compiled by N. Ramsing and J. Gundersen [65].

The equations are used to calculate the resuspension flux of MMHg and Hg^II^. The other variables are described in Table S11 and previous tables:

$k_{res}$ = $\frac{(E_{con}* SAw) * (1- f_{diss\_sed} )}{V_{sed}}$ [Equation S14]

$E_{con}$ = $E_{r}$ ** (*$t_{bs}- t_{cr}$*) ** 10^-5^ * *(60 * 60 * 24)* [Equation S15]

*f_diss_sed_*  = $\frac{1}{(1 + {10}^{kd}* sedsolids)}$ [Equation S16]

**Table S11:** The particulate Hg^II^ and MMHg resuspension to the overlaying water column.

| Parameter | Units | Description | Chesapeake Bay | Hudson River Estuary | Reference |
| --- | --- | --- | --- | --- | --- |
| *M_res_* | mol day^-1^ | Resuspension flux of Hg^II^ and MMHg to the benthic sediment | *Hg^II^_sed_p_ ** $k_{res}$*+ MMHg_sed_p_ ** $k_{res}$ | |  |
| $k_{res}$ | day^-1^ | Resuspension rate of particulate Hg^II^ and MMHg to the SBL reservoir | Equation S14 | |  |
| $E_{con}$ | m day^-1^ | Erosion rate | Equation S15 | | Ref. [51] |
| $E_{r}$ | kg m^-2^ s^-1^ | Erosion rate parameter | 3 x 10^-5^ | 5.5 x 10^-4^ | Ref. [66,67] |
| $t_{bs}$ | Pa | Bottom shear stress | 0.08 | 0.15 | Ref. [51,68] |
| $t_{cr}$ | Pa | Critical shear stress for erosion | 0.03 | 0.05 | Ref. [50,67] |
| *MMHg_sed_p_* | mol | Concentration of solid MMHg in the sediment reservoir | Table S1 | Table S2 |  |
| *Hg^II^_sed_p_* | mol | Concentration of solid Hg^II^ in the sediment reservoir | Table S1 | Table S2 |  |
| *f_diss_sed_* | unitless | Fraction of dissolved Hg^II^ and MMHg in the sediment | Equation S16 | |  |
| *kd* | L kg^-1^ | Benthic sediment partition coefficient of Hg^II^ and MMHg | 4.5 and 3.5 | 6.5 and 4.8 | Ref. [2,8] |

The equations used to calculate the burial flux in sediment. The other variables are described in Table S12 and previous tables:

*f_diss_sed_* = $\frac{1}{(1 + {10}^{kd}* sedsolids)}$ [Equation S17]

*k_bur_* = $\frac{{((B}_{rate\_mb} * SAs )/365.25 + (SAt * B_{rate_{{\_}_{it}}})/365.25)) *(1- f_{diss\_sed})}{V_{sed}}$ [Equation S18]

# **Table S12:** Hg chemical transformation in the sediment.

| Parameter | Units | | Description | Chesapeake Bay | Hudson River Estuary | Reference |  |
| --- | --- | --- | --- | --- | --- | --- | --- |
| *M_met_sed_* | mol day^-1^ | | Sediment methylation flux of Hg^II^ to MMHg | *Hg^II^_pw_* * *k_m_sed_* | |  |  |
| *M_dem_sed_* | mol day^-1^ | | Sediment demethylation flux of MMHg to  Hg^II^ | *MMHg_pw_* * *k_dm_sed_* | |  |  |
| *M_bur_* | mol day^-1^ | | Burial flux of Hg^II^ and MMHg | *k_bur_ * Hg^II^_sed_* + *k_bur_* * *MMHg_sed_* | |  |  |
| *k_m_sed_* | day^-1^ | | Average sediment methylation rate constant | *0.027* | | Ref. [2] |  |
| *k_dm_sed_* | day^-1^ | | Average sediment demethylation rate constant | 0.18 | | Ref. [69] |  |
| *k_bur_* | day^-1^ | Rate coefficients for sediment burial of Hg^II^ and MMHg | | Equation S18 | | Ref. [48] |  |
| *f_diss_sed_* | unitless | Fraction of Hg^II^ and MMHg in sediment in dissolved phase | | Equation S17 | |  |  |
| *Hg^II^_pw_* | pM | | Concentration of Hg^II^ in sediment porewater | Table S1 | Table S2 |  |  |
| *MMHg_pw_* | pM | | Concentration of MMHg in sediment porewater | Table S1 | Table S2 |  |  |
| *Hg^II^_sed_* | pM | | Concentration of Hg^II^ in sediment reservoir | Table S1 | Table S2 |  |  |
| *MMHg_sed_* | pM | | Concentration of MMHg in sediment reservoir | Table S1 | Table S2 |  |  |

# **Text S2:** Hg biogeochemical transformation

This section provides a comprehensive overview of all chemical parameters and the biogeochemical transformation processes incorporated in the model. A detailed summary of these biogeochemical transformations and their corresponding reaction rates can be found in **Tables S13-16**.

*Evasion:* The evasion flux of Hg^0^ and DMHg from the surface water through air-sea gas exchange was based on the parameterization of Nightingale *et al.* [70]; more information on the calculations is given in **Table S13-14**. The parameterization calculates the gas transfer velocity based on wind speed, temperature, and other factors influencing the exchange rate at the air-water interface. In these models, the gas transfer velocity is critical for determining the evasion flux of Hg^0^ and DMHg.

*Redox:* The model incorporates Hg redox dynamics via five key reaction pathways. Three principal mechanisms govern the reduction of Hg^II^ to Hg^0^: (1) chemical reduction, often referred to as dark reduction, (2) photo-reduction, and (3) biotic reduction. Reaction rates for these processes are derived from empirical studies reported in existing literature, as detailed in **Table S16**. The oxidation of Hg^0^ to Hg^II^ predominantly occurs through (4) chemical oxidation, known as dark oxidation, and (5) photo-oxidation. Photolysis rates are parameterized according to the intensity of photochemically active shortwave radiation at specific depths [48,71,72]. We limit the Hg pool available for photochemical activities to the euphotic depth to further constrain Hg redox flux. The rate of biotic reduction is scaled to net primary production [72], thereby accounting for the role of biological processes in modulating Hg redox cycling in the coastal environment.

*Methylation and demethylation in the water column:* We only consider the dissolved Hg pool when estimating the methylation flux because dissolved Hg species, particularly Hg^II^ and MMHg, are more bioavailable for bacteria uptake and subsequent methylation [73,74]. We use the average methylation rate of Hg^II^ to MMHg (0.0027 d^-1^) [75] and DMHg (1.3 × 10⁻⁵ d^-1^) [4]. The degradation rate of DMHg to MMHg (0.022 d^-1^) use was from recent studies of DMHg formation in the California Current [18]. The MMHg photodemethylation rate was estimated based on the parameterization of West *et al.* [76]. We constrained the photodemethylation process to the euphotic zone because it has been observed that the rates of photodemethylation rapidly decreased at depths (>0.5 m) below the surface [77]. At the base of the euphotic zone of coastal and marine surface waters, studies found a reduction in MeHg photochemical degradation due to the attenuation of UV-visible sunlight radiation, allowing biotic processes to be the most significant demethylating factor [78]. MMHg photodegradation rates depend on water constituents like thiol ligands that bind MeHg, making it reactive, and the process is influenced by factors like UV radiation, dissolved organic matter composition (DOM), and environmental conditions such as shading and hydrologic residence time [79,80]. All other demethylation rates of MeHg used in the model, including the biotic and dark demethylation, are further listed in **Table S16**.

*Methylation and demethylation in sediment*: We consider only two Hg species, Hg^II^ and MMHg, involved in the sediments' Hg biogeochemical transformation. The average methylation rate in benthic sediments is estimated at 0.027 d⁻¹, while the demethylation rate is 0.24 d⁻¹, based on research conducted along the North Atlantic continental margin [69,81]. To constrain the reservoir of Hg^II^ and MMHg available for both methylation and demethylation in the benthic sediments, only the fraction of Hg^II^ and MMHg present in the sediment porewater was used to calculate the total methylation flux [46]. This approach helps quantify Hg transformation within the sediment environment, providing insight into the cycling of MeHg in coastal ecosystems.

The set of equations is used to calculate the DMHg and Hg^0^ evasion from the surface water according to the method of Nightingale *et al.* [70]. The other variables are described in Tables S13 and S14:

$D_{DMHg}$ = $\frac{(7.4*{10}^{-8}) * {(\varphi* M_{w})}^{0.5}*(T_{w\_so}+273.15)}{(dv*{10}^{3}) * {V_{B\_DMHg}}^{0.6}}$ [Equation S19]

$k_{w}$ = *A **${u_{10}}^{2}$ ***${(\frac{{Sc}_{DMHg}}{600})}^{-0.5}$ *** $\frac{24}{100}$ [Equation S20]

*dv =* (1.88 x 10^-3^ – 0.04 x 10^-3^) * $T_{w\_so}$ [Equation S21]

${Ev}_{DMHg}$ = $k_{w}$ * ($C_{w\_DMHg}*1000- \frac{C_{a\_DMHg}}{H_{DMHg}}$) * SAw * $1^{-12}$ [Equation S22]

$D_{{Hg}^{0}}$ = $\frac{\left( 7.4*{10}^{-8} \right)* \left( \varphi* M_{w} \right)^{0.5}* (T_{w\_so}+273.15)}{(dv*{10}^{3}) * {V_{B\_{Hg}^{0}}}^{0.6}}$ [Equation S23]

${Ev}_{{Hg}^{0}}$ = $k_{w}$ * ($C_{w\_{Hg}^{0}}*1000- \frac{C_{a{\_Hg}^{0}}}{H_{{Hg}^{0}}}$) * SAw * $1^{-12}$ [Equation S24]

$H_{DMHg}$ = $e^{(\frac{-2512.43}{T_{w_{so}} + 273.15} + 7.27)}$ [Equation S25]

$u_{10}$ = 10.4 * $\frac{u}{(\log\left( W_{H} \right) + 8.1)}$ [Equation S26]

$H_{{Hg}^{0}}$ = $e^{(\frac{-2403.3}{T_{w_{so}} + 273.15} + 6.92)}$ [Equation S27]

# **Table S13:** Gas exchange of DMHg based on Nightingale *et al.* [70] calculations.

| Parameter | Units | Description | Chesapeake Bay | Hudson River Estuary | Reference |
| --- | --- | --- | --- | --- | --- |
| ${Ev}_{DMHg}$ | mol day^-1^ | DMHg evasion flux | Equation S22 | |  |
| $C_{a\_DMHg}$ | pmol m^-3^ | Concentration of DMHg in the air | 0.164 | | Ref. [82] |
| $k_{w}$ | m day^-1^ | Gas transfer velocity | Equation S20 | | Ref. [83] |
| ${Sc}_{DMHg}$ | unitless | Schmidt number for DMHg | $\frac{kv}{D_{DMHg}}$ | |  |
| $D_{DMHg}$ | cm^2^ s^-1^ | Diffusivity of DMHg | Equation S19 | | Ref. [84] |
| *kv* | m^2^ s^-1^ | Kinematic viscosity of the water | $\frac{dv}{\rho_{so}}$ | |  |
| $C_{w\_DMHg}$ | pM | Concentration of DMHg in MSL | Table S1 | Table S2 |  |
| *A* | unitless | Constant based on distribution of wind speeds over the ocean | 0.251 | | Ref. [85] |
| $H_{DMHg}$ | unitless | Dimensionless Henry's law constant | Equation S25 | | Ref. [86] |
| $u_{10}$ | m s^-1^ | Average wind speed at 10 m above water surface | Equation S26 | |  |
| $W_{H}$ | m | Height of wind speed measurement | 13 | 10 | Ref. [23,24] |
| *𝑢* | m s^-1^ | Average wind speed | 3-7 | 3-5 | Ref. [23,24] |
| *dv* | kg m^-1^ s^-1^ | Viscosity of water | Equation S21 | |  |
| $M_{w}$ | g mol^-1^ | Molecular weight of water | 18.01 | |  |
| $V_{B\_DMHg}$ | cm^3^ mol^-1^ | Molal volume of DMHg at its normal boiling temperature | 72.11 | |  |
| *φ* | unitless | Solvent association factor | 2.26 | | Ref. [87] |

**Table S14:** Gas exchange of Hg^0^ based on Nightingale *et al.* [70] calculations.

| Parameter | Units | Description | Chesapeake Bay | Hudson River Estuary | Reference |
| --- | --- | --- | --- | --- | --- |
| ${Ev}_{{Hg}^{0}}$ | mol day^-1^ | Hg^0^ evasion flux | Equation S24 | |  |
| $D_{{Hg}^{0}}$ | cm^2^ s^-1^ | Diffusivity of Hg^0^ | Equation S23 | | Ref. [84] |
| $C_{a{\_Hg}^{0}}$ | pmol m^-3^ | Concentration of Hg^0^ in the air | 9 | | Ref. [88] |
| $V_{B\_{Hg}^{0}}$ | cm^3^ mol^-1^ | Molal volume of Hg^0^ at its normal boiling temperature | 12.74 | |  |
| ${Sc}_{{Hg}^{0}}$ | unitless | Schmidt number for Hg^0^ | $\frac{kv}{D_{{Hg}^{0}}}$ | |  |
| $H_{{Hg}^{0}}$ | unitless | Dimensionless Henry's law constant | Equation S27 | | Ref. [89] |
| $V_{B\_{Hg}^{0}}$ | cm^3^ mol^-1^ | Molal volume of Hg^0^ at its normal boiling temperature | 12.74 | |  |
| $C_{w\_{Hg}^{0}}$ | pM | Concentration of Hg^0^ in MSL | Table S1 | Table S2 |  |

The equations used to calculate the shortwave radiation on the water column. The other variables are described in Table S15:

*RAD =* $\frac{1}{x_{2}- x_{1}}* \frac{R}{k}$ * [$e^{k * x_{1}}- e^{-k * x_{2}}]$ [Equation S28]

*k =* $k_{w}$+ $k_{DOC}$* *DOC* + $k_{Chla}$* *Chla* [Equation S29]

*KPAR = 0.92 + (0.079 * SPM_so ** 10^6^*) - (0.037 ** ${Sal\_}_{so}$*)* [Equation S30]

$Z_{eu}$ = $\frac{1.46}{(KPAR + \left( 0.12 * VSS \right))}$ [Equation S31]

# **Table S15:** Shortwave radiation in the water column.

| Parameter | Units | Description | Chesapeake Bay | Hudson River Estuary | Reference |
| --- | --- | --- | --- | --- | --- |
| *RAD* | W m^-2^ | Shortwave radiation at specific depth | Equation S28 | | Ref. [90] |
| $x_{1}$ | m | Surface depth | 0 | |  |
| $Z_{eu}$ | m | Euphotic depth | Equation S31 | | Ref. [91] |
| *k* | m^-1^ | Extinction coefficient for radiation | Equation S29 | |  |
| $k_{w}$ | m^-1^ | Extinction coefficient for water | 0.0145 | | Ref. [90] |
| $k_{Chla}$ | m^-1^ | Extinction coefficient for chlorophyll-a | 55 | | Ref. [90] |
| $k_{DOC}$ | mg L^-1^ | Extinction coefficient for dissolved organic carbon | 0.654 | | Ref. [90] |
| *R* | W m^-2^ | Average annual shortwave radiation at the water surface | 80 | | Ref. [92] |
| *DOC* | mg L^-1^ | Average concentration of dissolved organic carbon | 6 | 5.2 | Ref. [93,94] |
| *Chla* | mg L^-1^ | Average concentration of chlorophyll-a | 0.01 | 0.001-0.01 | Ref. [23,95] |
| *KPAR* | m^-1^ | Diffuse light attenuation coefficient | Equation S30 | | Ref. [91] |
| *VSS* | mg L^-1^ | Surface volatile suspended solids | 2.9 * *POC* | | Ref. [91] |
| *POC* | mg L^-1^ | Particulate organic carbon average concentration | 0.5 | 2 | Ref. [66,96] |
| *SPM_so* | mg L^-1^ | Concentration of particles in water in the SML | Table S8 | |  |

# **Table S16:** Hg chemical transformation in the water column.

| Parameter | Units | Description | Chesapeake Bay | Hudson River Estuary | Reference |
| --- | --- | --- | --- | --- | --- |
| *M_met1_HgII_* | mol day^-1^ | Methylation flux of Hg^II^ to MMHg | *Hg^II^_dis_* * *k_met_HgII_MMHg_* | |  |
| *M_met2_HgII_* | mol day^-1^ | Methylation flux of Hg^II^ to DMHg | *Hg^II^_dis_* * *k_met_HgII_DMHg_* | |  |
| *M_met_MMHg_* | mol day^-1^ | Methylation flux of MMHg to DMHg | *MMHg_dis_* * *k_met_MMHg_DMHg_* | |  |
| *M_dem_DMHg_* | mol day^-1^ | Demethylation flux of DMHg to  MMHg | *DMHg* * *k_dem_DMHg_MMHg_* | |  |
| *^+^M_dem_MMHg_* | mol day^-1^ | Demethylation flux of MMHg to  Hg^II^ | *MMHg_dis_* * (*k_phot_MMHg_HgII_dem_ + k_dark_dem_MMHg_*) | |  |
| *^+^M_ox_* | mol day^-1^ | Hg^0^ oxidation flux | *Hg^0^* * (*k_phot_ox_* + *k_dark_ox_*) | |  |
| *^+^M_red_* | mol day^-1^ | Hg^II^ reduction flux | *Hg^II^_dis_* * *F_red_* * (*k_phot_red_* + *k_bio_red_*) | |  |
| *k_met_HgII_MMHg_* | day^-1^ | Hg^II^ methylation rate to MMHg | 0.0027 | | Ref. [75] |
| *k_met_MMHg_DMHg_* | day^-1^ | MMHg methylation rate to DMHg | 4 x 10^-5^ | | Ref. [97] |
| *k_met_HgII_DMHg_* | day^-1^ | Hg^II^ methylation rate to DMHg | 1.3 x 10^-5^ | | Ref. [4] |
| *k_dem_DMHg_MMHg_* | day^-1^ | DMHg demethylation rate to MMHg | 0.022 | | Ref. [18] |
| *^+^k_dark_dem_MMHg_* | day^-1^ | Dark MMHg demethylation rate to Hg^II^ | *0.036* | | Ref. [98] |
| *k_phot_MMHg_HgII_dem_* | day^-1^ | MMHg to Hg^II^ Photo-demethylation rate constant | $(\frac{{Dg}_{MMHg}}{ALI})$* RAD | | Ref. [99] |
| *k_dark_ox_* | day^-1^ | Dark oxidation rate constant | 0.484 | | Ref. [100,101] |
| *k_bio_red_* | day^-1^ | Biotic reduction rate constant | 0.0450 * *NPP* | | Ref. [102] |
| *NPP* | gC m^-2^ day^-1^ | Average Net Primary Productivity | Table S8 | |  |
| *k_phot_ox_* | day^-1^ | Photo-oxidation rate constant | 0.57 * *RAD* | | Ref. [46] |
| *k_phot_red_* | day^-1^ | Photo-reduction rate constant | 0.16 * *RAD* | | Ref. [46] |
| *F_red_* | unitless | Fraction of reducible Hg^II^ | 0.4 | | Ref. [104] |
| *ALI* | W m^-2^ | Average light intensity | 581.7 | | Ref. [99] |
| ${Dg}_{MMHg}$ | day^-1^ | Degradation rate constant for MMHg | 0.54 | | Ref. [99] |
| *MMHg_dis_* | mol | Dissolved MMHg concentration in the reservoir | Table S1 | Table S2 |  |

# **Table S16:** Hg chemical transformation in the water column (Continued).

| Parameter | Units | Description | Chesapeake Bay | Hudson River Estuary | Reference |
| --- | --- | --- | --- | --- | --- |
| *Hg^II^_dis_* | mol | Dissolved Hg^II^ concentration in the reservoir | Table S1 | | Table S2 |

^+^The fraction of MMHg, Hg^0^, and Hg^II^ in the euphotic zone was used to calculate the photodecomposition, photooxidation, and photoreduction rate.

# **References**

[1] Mason RP, Lawson NM, Lawrence AL, Leaner JJ, Lee JG, Sheu G-R. Mercury in the Chesapeake Bay. Marine Chemistry 1999;65:77–96. https://doi.org/10.1016/S0304-4203(99)00012-2.

[2] Hollweg TA, Gilmour CC, Mason RP. Methylmercury production in sediments of Chesapeake Bay and the mid-Atlantic continental margin. Marine Chemistry 2009;114:86–101. https://doi.org/10.1016/j.marchem.2009.04.004.

[3] Bieser J, Amptmeijer DJ, Daewel U, Kuss J, Soerensen AL, Schrum C. The 3D biogeochemical marine mercury cycling model MERCY v2.0 – linking atmospheric Hg to methylmercury in fish. Geoscientific Model Development 2023;16:2649–88. https://doi.org/10.5194/gmd-16-2649-2023.

[4] Lehnherr I, St. Louis VL, Hintelmann H, Kirk JL. Methylation of inorganic mercury in polar marine waters. Nature Geosci 2011;4:298–302. https://doi.org/10.1038/ngeo1134.

[5] Mason RP, Sullivan KA. The distribution and speciation of mercury in the South and equatorial Atlantic. Deep Sea Research Part II: Topical Studies in Oceanography 1999;46:937–56. https://doi.org/10.1016/S0967-0645(99)00010-7.

[6] Munson KM, Lamborg CH, Swarr GJ, Saito MA. Mercury species concentrations and fluxes in the Central Tropical Pacific Ocean. Global Biogeochemical Cycles 2015;29:656–76. https://doi.org/10.1002/2015GB005120.

[7] Mason RP, Rolfhus KR, Fitzgerald WF. Mercury in the North Atlantic. Marine Chemistry 1998;61:37–53. https://doi.org/10.1016/S0304-4203(98)00006-1.

[8] Heyes A, Miller C, Mason RP. Mercury and methylmercury in Hudson River sediment: impact of tidal resuspension on partitioning and methylation. Marine Chemistry 2004;90:75–89. https://doi.org/10.1016/j.marchem.2004.03.011.

[9] Baeyens W, Leermakers M. Elemental mercury concentrations and formation rates in the Scheldt estuary and the North Sea. Marine Chemistry 1998;60:257–66. https://doi.org/10.1016/S0304-4203(97)00102-3.

[10] Benoit JM, Gilmour* CC, Mason RP, Riedel GS, Riedel GF. Behavior of mercury in the Patuxent River estuary. Biogeochemistry 1998;40:249–65. https://doi.org/10.1023/A:1005905700864.

[11] Schartup AT, Balcom PH, Soerensen AL, Gosnell KJ, Calder RSD, Mason RP, et al. Freshwater discharges drive high levels of methylmercury in Arctic marine biota. PROCEEDINGS OF THE NATIONAL ACADEMY OF SCIENCES OF THE UNITED STATES OF AMERICA 2015;112:11789–94. https://doi.org/10.1073/pnas.1505541112.

[12] Wall GR, Ingleston HH, Litten S. Calculating Mercury Loading to The Tidal Hudson River, New York, Using Rating Curve and Surrogate Methodologies. Water Air Soil Pollut 2005;165:233–48. https://doi.org/10.1007/s11270-005-5146-1.

[13] Lawson NM, Mason RP, Laporte J-M. The fate and transport of mercury, methylmercury, and other trace metals in chesapeake bay tributaries. Water Research 2001;35:501–15. https://doi.org/10.1016/S0043-1354(00)00267-0.

[14] Balcom PH, Hammerschmidt CR, Fitzgerald WF, Lamborg CH, O’Connor JS. Seasonal distributions and cycling of mercury and methylmercury in the waters of New York/New Jersey Harbor Estuary. Marine Chemistry 2008;109:1–17. https://doi.org/10.1016/j.marchem.2007.09.005.

[15] Bowman KL, Hammerschmidt CR, Lamborg CH, Swarr G. Mercury in the North Atlantic Ocean: The U.S. GEOTRACES zonal and meridional sections. Deep Sea Research Part II: Topical Studies in Oceanography 2015;116:251–61. https://doi.org/10.1016/j.dsr2.2014.07.004.

[16] Mason RP, Lawson NM, Sullivan KA. Atmospheric deposition to the Chesapeake Bay watershed—regional and local sources. Atmospheric Environment 1997;31:3531–40. https://doi.org/10.1016/S1352-2310(97)00207-0.

[17] Felton D, Civerolo K. Air Monitoring Plan for Establishing an Ambient Mercury Baseline for New York State, New York State Dept. of Environmental Conservation; 2011.

[18] Adams HM, Cui X, Lamborg CH, Schartup AT. Dimethylmercury as a Source of Monomethylmercury in a Highly Productive Upwelling System. Environ Sci Technol 2024;58:10591–600. https://doi.org/10.1021/acs.est.4c01112.

[19] Mason RP, Rolfhus KR, Fitzgerald WF. Mercury in the North Atlantic. Marine Chemistry 1998;61:37–53. https://doi.org/10.1016/S0304-4203(98)00006-1.

[20] Munson KM, Lamborg CH, Boiteau RM, Saito MA. Dynamic mercury methylation and demethylation in oligotrophic marine water. Biogeosciences 2018;15:6451–60. https://doi.org/10.5194/bg-15-6451-2018.

[21] Mason RP, Lawson NM, Sheu GR. Annual and seasonal trends in mercury deposition in Maryland. Atmospheric Environment 2000;34:1691–701. https://doi.org/10.1016/S1352-2310(99)00428-8.

[22] Hinson KE, Friedrichs MAM, St-Laurent P, Da F, Najjar RG. Extent and Causes of Chesapeake Bay Warming. JAWRA Journal of the American Water Resources Association 2022;58:805–25. https://doi.org/10.1111/1752-1688.12916.

[23] New York City Department of Environmental Protection. Harbor Water Quality, 2015. Data accessed from NYC website: https://data.cityofnewyork.us/widgets/5uug-f49n; accessed 06/04/2024. n.d.

[24] Chesapeake Bay National Estuarine Research Reserve in Virginia, Virginia Institute of Marine Science (CBNERR-VA VIMS), 2024. Virginia Estuarine and Coastal Observing System (VECOS). Data accessed from VECOS website: http://vecos.vims.edu; accessed 06/04/2024. n.d.

[25] Brosnan TM, O’Shea ML. Long-Term Improvements in Water Quality Due to Sewage Abatement in the Lower Hudson River. Estuaries 1996;19:890–900. https://doi.org/10.2307/1352305.

[26] Chesapeake Bay Program (2024), CBP Water Quality Database (1984-Present) 2024 Edition n.d.

[27] Jiang L, Xia M. Dynamics of the Chesapeake Bay outflow plume: Realistic plume simulation and its seasonal and interannual variability. Journal of Geophysical Research: Oceans 2016;121:1424–45. https://doi.org/10.1002/2015JC011191.

[28] Kearney MS, Rogers AS, Townshend JRG, Rizzo E, Stutzer D, Stevenson JC, et al. Landsat imagery shows decline of coastal marshes in Chesapeake and Delaware Bays. Eos, Transactions American Geophysical Union 2002;83:173–8. https://doi.org/10.1029/2002EO000112.

[29] NOAA. Shoreline Data Rescue Project of Hudson River, NY, EC5C01 | InPort n.d. https://www.fisheries.noaa.gov/inport/item/63633 (accessed July 31, 2025).

[30] Levinton JS, Waldman JR, editors. The Hudson River Estuary. Cambridge: Cambridge University Press; 2006. https://doi.org/10.1017/CBO9780511550539.

[31] Lee SB, Li M, Zhang F. Impact of sea level rise on tidal range in Chesapeake and Delaware Bays. Journal of Geophysical Research: Oceans 2017;122:3917–38. https://doi.org/10.1002/2016JC012597.

[32] Center for Operational Oceanographic Products and Services (CO-OPS), 2024. NOAA Tide Predictions. National Oceanic and Atmospheric Administration (NOAA). Accessed July 10, 2024. Available at: https://tidesandcurrents.noaa.gov/tide_predictions.html. n.d.

[33] Tabak NM, Laba M, Spector S. Simulating the Effects of Sea Level Rise on the Resilience and Migration of Tidal Wetlands along the Hudson River. PLOS ONE 2016;11:e0152437. https://doi.org/10.1371/journal.pone.0152437.

[34] Bilkovic DM, Mitchell MM, Havens KJ, Hershner CH. Chapter 15 - Chesapeake Bay. In: Sheppard C, editor. World Seas: an Environmental Evaluation (Second Edition), Academic Press; 2019, p. 379–404. https://doi.org/10.1016/B978-0-12-805068-2.00019-X.

[35] WillyWeather. Hudson River - Chelsea Piers 2024.

[36] U.S. Geological Survey. Water Data for the Chesapeake Bay, accessed April 12, 2024, at https://www.usgs.gov/centers/chesapeake-bay-activities/science/freshwater-flow-chesapeake-bay. n.d.

[37] U.S. Geological Survey. Water Data for the Nation, accessed March 25, 2024, at http://waterdata.usgs.gov/nwis/. n.d.

[38] U.S. Geological Survey. Water Data for the Hudson River at Green Island NY, accessed July 14, 2024, at https://waterdata.usgs.gov/monitoring-location/01358000/#dataTypeId=continuous-00065-0&period=P7D&showMedian=false. n.d.

[39] Laurent A, Fennel K, Wilson R, Lehrter J, Devereux R. Parameterization of biogeochemical sediment–water fluxes using in situ measurements and a diagenetic model. Biogeosciences 2016;13:77–94. https://doi.org/10.5194/bg-13-77-2016.

[40] Woodruff JD, Geyer WR, Sommerfield CK, Driscoll NW. Seasonal variation of sediment deposition in the Hudson River estuary. Marine Geology 2001;179:105–19. https://doi.org/10.1016/S0025-3227(01)00182-7.

[41] Lemagie E, Lerczak J. A Comparison of Bulk Estuarine Turnover Timescales to Particle Tracking Timescales Using a Model of the Yaquina Bay Estuary. Estuaries and Coasts 2014;38. https://doi.org/10.1007/s12237-014-9915-1.

[42] Sheldon JE, Alber M. The calculation of estuarine turnover times using freshwater fraction and tidal prism models: A critical evaluation. Estuaries and Coasts 2006;29:133–46. https://doi.org/10.1007/BF02784705.

[43] Dyer, K.R., 1973. Estuaries: A Physical Introduction. Wiley, London. n.d.

[44] Ralston DK, Geyer WR, Lerczak JA. Subtidal Salinity and Velocity in the Hudson River Estuary: Observations and Modeling 2008. https://doi.org/10.1175/2007JPO3808.1.

[45] Gill GA, Bloom NS, Cappellino S, Driscoll CT, Dobbs C, Mcshea L, et al. Sediment-water fluxes of mercury in Lavaca Bay, Texas. Environmental Science and Technology 1999;33:663–9. https://doi.org/10.1021/es980380c.

[46] Soerensen AL, Jacob DJ, Schartup AT, Fisher JA, Lehnherr I, St. Louis VL, et al. A mass budget for mercury and methylmercury in the Arctic Ocean: ARCTIC OCEAN HG AND MEHG MASS BUDGET. Global Biogeochem Cycles 2016;30:560–75. https://doi.org/10.1002/2015GB005280.

[47] Nasiha HJ, Shanmugam P. Estimation of settling velocity of sediment particles in estuarine and coastal waters. Estuarine, Coastal and Shelf Science 2018;203:59–71. https://doi.org/10.1016/j.ecss.2018.02.001.

[48] Sunderland EM, Dalziel J, Heyes A, Branfireun BA, Krabbenhoft DP, Gobas FAPC. Response of a macrotidal estuary to changes in anthropogenic mercury loading between 1850 and 2000. Environ Sci Technol 2010;44:1698–704. https://doi.org/10.1021/es9032524.

[49] Moriarty JM, Friedrichs MAM, Harris CK. Seabed Resuspension in the Chesapeake Bay: Implications for Biogeochemical Cycling and Hypoxia. Estuaries and Coasts 2021;44:103–22. https://doi.org/10.1007/s12237-020-00763-8.

[50] Cerco CF, Kim S-C, Noel MR. Management modeling of suspended solids in the Chesapeake Bay, USA. Estuarine, Coastal and Shelf Science 2013;116:87–98. https://doi.org/10.1016/j.ecss.2012.07.009.

[51] Sanford LP, Maa JP-Y. A unified erosion formulation for fine sediments. Marine Geology 2001;179:9–23. https://doi.org/10.1016/S0025-3227(01)00201-8.

[52] National Oceanic and Atmospheric Administration (NOAA), 2024: Chesapeake Bay Operational Forecast System (CBOFS), accessed 06 April 2024, https://tidesandcurrents.noaa.gov/ofs/cbofs/cbofs.html n.d.

[53] Sirois DL, Fredrick SW. Phytoplankton and primary production in the lower Hudson River estuary. Estuarine and Coastal Marine Science 1978;7:413–23. https://doi.org/10.1016/0302-3524(78)90118-4.

[54] Son S, Wang M, Harding LW. Satellite-measured net primary production in the Chesapeake Bay. Remote Sensing of Environment 2014;144:109–19. https://doi.org/10.1016/j.rse.2014.01.018.

[55] Li J, Reardon P, McKinley JP, Joshi SR, Bai Y, Bear K, et al. Water column particulate matter: A key contributor to phosphorus regeneration in a coastal eutrophic environment, the Chesapeake Bay. Journal of Geophysical Research: Biogeosciences 2017;122:737–52. https://doi.org/10.1002/2016JG003572.

[56] Ralston DK, Geyer WR, Lerczak JA. Subtidal Salinity and Velocity in the Hudson River Estuary: Observations and Modeling 2008. https://doi.org/10.1175/2007JPO3808.1.

[57] Saenger C, Cronin TM, Willard D, Halka J, Kerhin R. Increased Terrestrial to Ocean Sediment and Carbon Fluxes in the Northern Chesapeake Bay Associated With Twentieth Century Land Alteration. Estuaries and Coasts 2008;31:492–500. https://doi.org/10.1007/s12237-008-9048-5.

[58] Palinkas CM, Bolton MC, Staver LW. Long-term performance and impacts of living shorelines in mesohaline Chesapeake Bay. Ecological Engineering 2023;190:106944. https://doi.org/10.1016/j.ecoleng.2023.106944.

[59] Dellapenna TM, Kuehl SA, Schaffner LC. Ephemeral deposition, seabed mixing and fine-scale strata formation in the York River estuary, Chesapeake Bay. Estuarine, Coastal and Shelf Science 2003;58:621–43. https://doi.org/10.1016/S0272-7714(03)00174-4.

[60] Li M, Zhong L, Boicourt WC. Simulations of Chesapeake Bay estuary: Sensitivity to turbulence mixing parameterizations and comparison with observations. Journal of Geophysical Research: Oceans 2005;110. https://doi.org/10.1029/2004JC002585.

[61] Stenström P. Hydraulics and mixing in the Hudson River estuary: A numerical model study of tidal variations during neap tide conditions. Journal of Geophysical Research: Oceans 2004;109. https://doi.org/10.1029/2003JC001954.

[62] Gibbs RJ, Jha PK, Chakrapani GJ. Sediment particle size in the Hudson River Estuary. Sedimentology 1994;41:1063–8. https://doi.org/10.1111/j.1365-3091.1994.tb01441.x.

[63] San Francisco Bay/Estuary (SFBE) | Ramsar Sites Information Service n.d. https://rsis.ramsar.org/ris/2097 (accessed May 6, 2024).

[64] US EPA O. Understanding Variation in Partition Coefficient, Kd, Values 2015. https://www.epa.gov/radiation/understanding-variation-partition-coefficient-kd-values (accessed March 20, 2023).

[65] N. Ramsing and J. Gundersen. Seawater and Gases, Unisense, accessed on 08 April 2024, https://unisense.com/wp-content/uploads/2021/10/Seawater-Gases-table.pdf n.d.

[66] Moriarty JM, Friedrichs MAM, Harris CK. Seabed Resuspension in the Chesapeake Bay: Implications for Biogeochemical Cycling and Hypoxia. Estuaries and Coasts 2021;44:103–22. https://doi.org/10.1007/s12237-020-00763-8.

[67] Ralston DK, Geyer WR, Warner JC. Bathymetric controls on sediment transport in the Hudson River estuary: Lateral asymmetry and frontal trapping. Journal of Geophysical Research: Oceans 2012;117. https://doi.org/10.1029/2012JC008124.

[68] Ralston DK, Geyer WR, Warner JC. Bathymetric controls on sediment transport in the Hudson River estuary: Lateral asymmetry and frontal trapping. Journal of Geophysical Research: Oceans 2012;117. https://doi.org/10.1029/2012JC008124.

[69] Heyes A, Mason RP, Kim E-H, Sunderland E. Mercury methylation in estuaries: Insights from using measuring rates using stable mercury isotopes. Marine Chemistry 2006;102:134–47. https://doi.org/10.1016/j.marchem.2005.09.018.

[70] Nightingale PD, Malin G, Law CS, Watson AJ, Liss PS, Liddicoat MI, et al. In situ evaluation of air-sea gas exchange parameterizations using novel conservative and volatile tracers. Global Biogeochemical Cycles 2000;14:373–87. https://doi.org/10.1029/1999GB900091.

[71] Schwarzenbach RP, Gschwend PM, Imboden DM. Environmental organic chemistry. 2. ed. New York: Wiley; 2003.

[72] Whalin L, Kim E-H, Mason R. Factors influencing the oxidation, reduction, methylation and demethylation of mercury species in coastal waters. Marine Chemistry 2007;107:278–94. https://doi.org/10.1016/j.marchem.2007.04.002.

[73] Hsu-Kim H, Kucharzyk KH, Zhang T, Deshusses MA. Mechanisms Regulating Mercury Bioavailability for Methylating Microorganisms in the Aquatic Environment: A Critical Review. Environ Sci Technol 2013;47:2441–56. https://doi.org/10.1021/es304370g.

[74] Mazrui NM, Jonsson S, Thota S, Zhao J, Mason RP. Enhanced availability of mercury bound to dissolved organic matter for methylation in marine sediments. Geochimica et Cosmochimica Acta 2016;194:153. https://doi.org/10.1016/j.gca.2016.08.019.

[75] Despins MC, Mason RP, Aguilar-Islas AM, Lamborg CH, Hammerschmidt CR, Newell SE. Linked mercury methylation and nitrification across oxic subpolar regions. Frontiers in Environmental Chemistry 2023;4.

[76] West J, Gindorf S, Jonsson S. Photochemical Degradation of Dimethylmercury in Natural Waters. Environ Sci Technol 2022;56:5920–8. https://doi.org/10.1021/acs.est.1c08443.

[77] Eckley CS, Luxton TP, Knightes CD, Shah V. Methylmercury Production and Degradation under Light and Dark Conditions in the Water Column of the Hells Canyon Reservoirs, USA. Environmental Toxicology and Chemistry 2021;40:1827–37. https://doi.org/10.1002/etc.5041.

[78] Monperrus M, Tessier E, Amouroux D, Leynaert A, Huonnic P, Donard OFX. Mercury methylation, demethylation and reduction rates in coastal and marine surface waters of the Mediterranean Sea. Marine Chemistry 2007;107:49–63. https://doi.org/10.1016/j.marchem.2007.01.018.

[79] Fleck JA, Gill G, Bergamaschi BA, Kraus TEC, Downing BD, Alpers CN. Concurrent photolytic degradation of aqueous methylmercury and dissolved organic matter. Science of The Total Environment 2014;484:263–75. https://doi.org/10.1016/j.scitotenv.2013.03.107.

[80] Zhang T, Hsu-Kim H. Photolytic degradation of methylmercury enhanced by binding to natural organic ligands. Nat Geosci 2010;3:473–6. https://doi.org/10.1038/ngeo892.

[81] Hollweg TA, Gilmour CC, Mason RP. Mercury and methylmercury cycling in sediments of the mid-Atlantic continental shelf and slope. Limnology and Oceanography 2010;55:2703–22. https://doi.org/10.4319/lo.2010.55.6.2703.

[82] Baya PA, Gosselin M, Lehnherr I, St Louis VL, Hintelmann H. Determination of monomethylmercury and dimethylmercury in the Arctic marine boundary layer. Environ Sci Technol 2015;49:223–32. https://doi.org/10.1021/es502601z.

[83] Wanninkhof R. Relationship between wind speed and gas exchange over the ocean. Journal of Geophysical Research: Oceans 1992;97:7373–82. https://doi.org/10.1029/92JC00188.

[84] Wilke CR, Chang P. Correlation of diffusion coefficients in dilute solutions. AIChE Journal 1955;1:264–70. https://doi.org/10.1002/aic.690010222.

[85] Sweeney C, Gloor E, Jacobson AR, Key RM, McKinley G, Sarmiento JL, et al. Constraining global air-sea gas exchange for CO2 with recent bomb 14C measurements. Global Biogeochemical Cycles 2007;21. https://doi.org/10.1029/2006GB002784.

[86] Lindqvist O, Rodhe H. Atmospheric mercury—a review*. Tellus B 1985;37B:136–59. https://doi.org/10.1111/j.1600-0889.1985.tb00062.x.

[87] Hayduk W, Laudie H. Prediction of diffusion coefficients for nonelectrolytes in dilute aqueous solutions. AIChE Journal 1974;20:611–5. https://doi.org/10.1002/aic.690200329.

[88] Laurier F, Mason R. Mercury concentration and speciation in the coastal and open ocean boundary layer. Journal of Geophysical Research: Atmospheres 2007;112. https://doi.org/10.1029/2006JD007320.

[89] Andersson ME, Gårdfeldt K, Wängberg I, Strömberg D. Determination of Henry’s law constant for elemental mercury. Chemosphere 2008;73:587–92. https://doi.org/10.1016/j.chemosphere.2008.05.067.

[90] Schwarzenbach RP, Gschwend PM, Imboden DM. Environmental organic chemistry. 2. ed. New York: Wiley; 2003.

[91] Turner JS, St-Laurent P, Friedrichs MAM, Friedrichs CT. Effects of reduced shoreline erosion on Chesapeake Bay water clarity. Science of The Total Environment 2021;769:145157. https://doi.org/10.1016/j.scitotenv.2021.145157.

[92] Jin Z, Charlock TP, Rutledge K, Cota G, Kahn R, Redemann J, et al. Radiative Transfer Modeling for the CLAMS Experiment. Journal of the Atmospheric Sciences 2005;62:1053–71. https://doi.org/10.1175/JAS3351.1.

[93] Findlay SE. Increased carbon transport in the Hudson River: unexpected consequence of nitrogen deposition? Frontiers in Ecology and the Environment 2005;3:133–7. https://doi.org/10.1890/1540-9295(2005)003[0133:ICTITH]2.0.CO;2.

[94] Henderson R, Bukaveckas PA. Factors Governing Light Attenuation in Upper Segments of the James and York Estuaries and Their Influence on Primary Producers. Estuaries and Coasts 2022;45:470–84. https://doi.org/10.1007/s12237-021-00983-6.

[95] Yu X, Shen J. A data-driven approach to simulate the spatiotemporal variations of chlorophyll-a in Chesapeake Bay. Ocean Modelling 2021;159:101748. https://doi.org/10.1016/j.ocemod.2020.101748.

[96] Findlay S, Pace M, Lints D. Variability and Transport of Suspended Sediment, Particulate and Dissolved Organic Carbon in the Tidal Freshwater Hudson River. Biogeochemistry 1991;12:149–69.

[97] Kanzler CR, Lian P, Trainer EL, Yang X, Govind N, Parks JM, et al. Emerging investigator series: methylmercury speciation and dimethylmercury production in sulfidic solutions. Environ Sci: Processes Impacts 2018;20:584–94. https://doi.org/10.1039/C7EM00533D.

[98] Soerensen AL, Schartup AT, Skrobonja A, Bouchet S, Amouroux D, Liem-Nguyen V, et al. Deciphering the Role of Water Column Redoxclines on Methylmercury Cycling Using Speciation Modeling and Observations From the Baltic Sea. Global Biogeochemical Cycles 2018;32:1498–513. https://doi.org/10.1029/2018GB005942.

[99] West J, Gindorf S, Jonsson S. Photochemical Degradation of Dimethylmercury in Natural Waters. Environ Sci Technol 2022;56:5920–8. https://doi.org/10.1021/acs.est.1c08443.

[100] Lalonde JD, Amyot M, Orvoine J, Morel FMM, Auclair J-C, Ariya PA. Photoinduced oxidation of Hg0(aq) in the waters from the St. Lawrence estuary. Environ Sci Technol 2004;38:508–14. https://doi.org/10.1021/es034394g.

[101] Lalonde JD, Amyot M, Kraepiel AML, Morel FMM. Photooxidation of Hg(0) in Artificial and Natural Waters. Environ Sci Technol 2001;35:1367–72. https://doi.org/10.1021/es001408z.

[102] Soerensen AL, Sunderland EM, Holmes CD, Jacob DJ, Yantosca RM, Skov H, et al. An Improved Global Model for Air-Sea Exchange of Mercury: High Concentrations over the North Atlantic. Environ Sci Technol 2010;44:8574–80. https://doi.org/10.1021/es102032g.

[103] Guentzel JL, Powell RT, Landing WM, Mason RP. Mercury associated with colloidal material in an estuarine and an open-ocean environment. Marine Chemistry 1996;55:177–88. https://doi.org/10.1016/S0304-4203(96)00055-2.

[104] Kuss J, Wasmund N, Nausch G, Labrenz M. Mercury Emission by the Baltic Sea: A Consequence of Cyanobacterial Activity, Photochemistry, And Low-Light Mercury Transformation. Environ Sci Technol 2015;49:11449–57. https://doi.org/10.1021/acs.est.5b02204.
